# Integrated LSPR Biosensing Signal Processing Strategy and Visualization Implementation

**DOI:** 10.3390/mi15050631

**Published:** 2024-05-08

**Authors:** Mixing Zhou, Zhaoxin Geng

**Affiliations:** 1 School of Information Engineering, Minzu University of China, Beijing 100081, China; 21301983@muc.edu.cn; 2 Key Laboratory of Ethnic Language Intelligent Analysis and Security Governance of MOE, Minzu University of China, Beijing 100081, China

**Keywords:** spectral analysis, sensitivity, figure of merit, LSPR biosensor, integrated software

## Abstract

The LSPR biosensor chip is a groundbreaking tool popular in laboratory settings for identifying disease markers. However, its use in clinical environments is not as widespread. One notable gap is the lack of a universal signal processing tool for LSPR biosensing. To escalate its precision, there is an emerging need for software that not only optimizes signal processing but also incorporates self-verification functionalities within LSPR biochemical sensors. Enter the visual LSPR sensor software—an innovative platform that processes real-time transmission or reflection spectra. This advanced software adeptly captures the nuanced structural changes at the nanostructure interface prompted by environmental fluctuations. It diligently records and computes a suite of parameters, including the resonance wavelength shift, full width at half maximum, sensitivity, and quality factor. These features empower users to tailor processing algorithms for each data capture session. Transcending traditional instruments, this method accommodates a multitude of parameters and ensures robust result validation while tactfully navigating nanostructure morphology complexities. Forsaking third-party tool dependencies, the software tackles challenges of precision and cost-effectiveness head-on, heralding a significant leap forward in nanophotonics, especially for high-throughput LSPR biosensing applications. This user-centric innovation marks substantial progress in biochemical detection. It is designed to serve both researchers and practitioners in the field of nanophotonic sensing technology, simplifying complexity while enhancing reliability and efficiency.

## 1. Introduction

In the field of conventional localized surface plasmon resonance (LSPR) biosensors, the foundation lies in receptor-functionalized gold nanoparticles (AuNPs), providing specific binding capabilities to target molecules. Upon the attachment of the target molecule to the nanoparticle surface, a consequential redshift and attenuation of LSPR peaks occur [1]. To elevate the sensitivity of LSPR biosensors, diverse strategies come into play, which include the optimization of metal nanostructures, substrate materials, and modification methods of the interface [2]. However, it is worth noting that these approaches may entail high processing costs and yield lower results [3]. To solve this problem, the detection limit of local surface plasmon resonance biosensors is further intricately tied to the processing method of sensing signals. Numerous researchers optimize the data processing methods for LSPR peak wavelength through several key strategies: the first is advanced spectral analysis algorithms, such as polynomial curve fitting [4,5], wavelet transforms [6,7], fixed baseline algorithm [8], dynamic baseline algorithm [9], constant algorithm [10], and so on. The second is adaptive data filtering, such as Savitzky–Golay polynomial filter [11,12], wavelet transform [13], singular value decomposition [14], morphological filtering [15], and so on. The third is real-time monitoring and feedback [4], which enables the dynamic adjustment of data processing parameters, ensuring accurate determination of LSPR spectral resonant peak wavelengths under varying conditions.

Various software is available for working with surface plasmon resonance (SPR) or LSPR spectral signals; however, traditional LSPR or SPR signal processing software still has many problems to solve. Typically, the raw data generated by LSPR testing devices contain noise and outliers, necessitating preprocessing before visualization. Unfortunately, the processing of SPR/LSPR data is frequently a manual and time-consuming task. This is evident in software solutions like Anabel [16] and Sim-SPR [17]. Some applications include an important feature in their products: visualization of raw signal processing and analysis. Dahl et al. developed a unified web-based SPR data processing and analysis platform; however, it was adapted to be compatible only with Biacore SPR devices [18]. Krämer et al. also created an online, open-source SPR analysis software; however, the user interface is still difficult for novice users to understand due to the complexity of the data processing functions [16]. Some inexpensive SPR devices, such as the NanoSPR, generally do not come with user-friendly software and are not equipped with automatic signal processing. Some software could only be used for the analysis of static spectral files, such as a software called NANOPTICS developed by Marco S. et al. [19]. The real-time software system could monitor and provide feedback on the changes in LSPR signals so that users could quickly obtain experimental dynamic information. Compared to batch processing or periodic analysis of other systems, real-time could help to capture the interaction between the sample and the sensor more sensitively, improving the timeliness and efficiency of the experimental process. In medical applications, the capability of detecting multiple parameters at a specific point is crucial. This detection process should encompass essential features like small size, label-free sensing, real-time monitoring, and high sensitivity. The ability to detect multiple parameters not only addresses these requirements but also facilitates the correlation of related parameters, thereby enhancing accuracy in diagnosis [20].

Introducing a versatile LSRP sensing software system for real-time monitoring of biomolecular interactions, we conduct multiple detection point measurements under various reagent conditions. Employing multi-spectral testing methods allows for the simultaneous acquisition of multiple measurement data, enabling self-calibration or self-reference during testing or data processing. The integrated LSPR sensing automation software system excels in rapid, multiplex, and multi-parallel continuous biomarker detection, with wide-ranging applications such as immune status monitoring, cancer diagnosis, and prognosis. Leveraging the real-time, dynamic, and multi-point analyte detection capabilities of the software system, the LSPR nanoplasmonic sensor demonstrates substantial potential for integration into high-throughput or multiplexed immunoassays. This application holds the promise of simplifying disease diagnosis and advancing biomedical research through a straightforward and cost-effective platform. LSPR sensing principle and signal acquisition on the integrated visual software.

## 2. LSPR Sensing Principles on the Integrated Visual Software

### 2.1. Comparison of Different LSPR Sensing Parameters

LSPR biosensing technology stands as a potent analytical tool, offering insights into the microscopic level through its sensitive response to local permittivity changes on the surface. Within this realm, essential LSPR sensing parameters, including resonance wavelength, centroid, and center wavelength, play pivotal roles in comprehending and interpreting the sensor response. The article subsequently explores the significance of these parameters, highlighting their crucial role in advancing LSPR technology.

Taking LSPR extinction spectra as an example. The wavelength at which the extinction spectra of the noble metal nanostructure reach their peak is called the resonance wavelength ($\lambda_{max}$). $\lambda_{max}$ and the amplitude of the peak ($I_{max}$) both indicate molecular binding events. When molecules bind to the surface of the metal nanoparticles, they alter the local refractive index, causing a redshift in the resonance peak wavelength and amplitude. $\lambda_{max}$ and $I_{max}$ also indicate analyte concentration. The magnitude of the shift in resonance peak wavelength is often proportional to the concentration of the analyte binding to the surface. $\lambda_{max}$ could be applied to real-time detection. The resonance peak wavelength could be monitored in real time, allowing for the continuous observation of molecular interactions. It also indicates the shift in resonance wavelength coupled with molecular binding events. Centroid wavelength $\left(\lambda_{c}\right)$ defines the position of the spectral center of mass. $\lambda_{c}$ is often used when the shape of the resonance peaks is irregular. Focusing on the centroid mitigates the effects of noise or fluctuations at the edges of the resonance peak. The centroid provides a more stable measure of the central position, which could be particularly beneficial in low signal-to-noise situations. Another commonly used parameter is the center wavelength ($\lambda_{s}$), which is defined as the center of the full width at half maximum (*FWHM*). *FWHM* gives information about the width at half amplitude of the peak, and full width at quarter maximum (*FWQM*) gives information about the width of a line shape at half of its maximum amplitude. A smaller *FWHM* corresponds to a sharper resonance peak. A sharp peak indicates a narrow range of wavelengths at which the plasmon resonance occurs. This sharpness is desirable in sensing applications because it allows for a more precise determination of the resonance wavelength. If the refractive index of the surrounding medium increases, the extinction spectra are redshifted, and the resonance wavelength increases; at this point, the shifting resonance wavelength $∆\lambda_{max}$ is expressed as:

(1) $$∆\lambda_{max}=\lambda_{max}’-\lambda_{max}$$

where $∆\lambda_{max}$ is often used for monitoring changes in the absorption curve maximum or minimum as the local dielectric environment changes due to analyte adsorption. Similarly, observing the change in resonance wavelength from different perspectives, the change in *FWHM*, *FWQM*, $\lambda_{c}$, $\lambda_{s}$ is defined, as shown in the Supplementary Materials, which is monitored in sensing processes.

In assessing sensor performance, the utilization of multiple resonance characterization parameters is driven by their ability to furnish a diverse range of information regarding the sensor’s response. This facilitates a more thorough and precise delineation of the sensor’s performance characteristics. By monitoring the position of the resonance peak, more spectral information could be obtained from multiple spectral sensing parameters to improve the accuracy and sensitivity of LSPR sensing detection by monitoring the $\lambda_{max}$, $\lambda_{c}$, $\lambda_{s}$, $I_{max}$, FWHM and other parameters of the resonance peaks. The combination of multiple parameters provides a more complete picture of how the sensor performs in different aspects. For example, the resonance center wavelength can provide information about the sensitivity and selectivity of the sensor, while the amplitude can provide the sensor’s response to concentration. When designing and optimizing sensor performance, the use of multiple parameters helps to consider different aspects of the need, making the sensor more flexible and reliable in practical applications. In the field of integrated visualization software systems for LSPR spectra sensing, parameters such as sensitivity (S), figure of merit (FOM), spectral resolution (R), and limit of detection (LOD) play pivotal roles. Ref. [21] Demonstrates that sensitivity is crucial for detecting subtle changes in the refractive index of the surrounding medium, enhancing the system’s ability to discern minute variations. FOM reflects the efficiency and precision of the LSPR phenomenon, directly impacting the reliability of the sensor. Spectral resolution is vital for distinguishing closely spaced resonances, contributing to the system’s capability to resolve complex spectra. LOD, on the other hand, defines the lowest analyte concentration detectable by the system, influencing its practical utility in diverse applications. In summary, optimizing sensitivity, FOM, spectral resolution, and detection limits in the context of integrated LSPR spectra sensing software is essential for enhancing the overall performance and applicability of the system in various analytical scenarios. Specific details about these parameters are provided in the Supplementary Materials.

Similarly, key statistical parameters such as signal-to-noise ratio (*SNR*), standard deviation (σ), variance ($\sigma^{2}$), and coefficient of variation (*CV*) play critical roles. Ref. [22] Demonstrates that *SNR* is fundamental for distinguishing the signal of interest from background noise, ensuring the reliability of the sensor’s measurements. σ and $\sigma^{2}$ provide insights into the dispersion and stability of the data, influencing the precision and consistency of the LSPR system. *CV* offers a normalized measure of data variability, aiding in the assessment of relative variation across different datasets. Altogether, optimizing *SNR*, minimizing standard deviation and variance, and maintaining an appropriate coefficient of variation are critical for enhancing the robustness and accuracy of the integrated LSPR spectroscopic sensing software system. Specific details about these parameters are in the Supplementary Materials.

### 2.2. Multiple Resonance Peak Characterization Algorithms Integrated in the Visual Software

Based on LSPR spectra preprocessing (specific details about the spectra preprocessing are provided in the Supplementary Materials), we optimized the signal processing algorithm to further enhance the interpretation ability of the LSPR sensor output.

The determination of the wavelength of the LSPR is a crucial task in various applications, and several algorithms are commonly employed for this purpose. The centroid algorithm with a fixed baseline calculates the centroid of the LSPR peak relative to a fixed baseline, offering simplicity but sensitivity to baseline variability. The polynomial fit algorithm fits a polynomial function to the LSPR spectra, providing a smooth representation but requiring careful selection of the polynomial degree. The dynamic baseline algorithm adapts the baseline dynamically, enhancing robustness to baseline variations. The constant reflectance algorithm detects changes in spectral signal intensity at a fixed wavelength, offering simplicity with sensitivity to noise. The multi-scale continuous wavelet transform algorithm employs wavelet transforms at multiple scales, capturing various features and exhibiting less sensitivity to noise. The quadruple central moments algorithm [19] is a statistical analysis of LSPR spectra with normalized spectral distributions. It was performed to compare different sensing platforms in this method and to compute the four statistical central moments of the spectral distributions. The choice of algorithm depends on the specific characteristics of the LSPR spectra and the application’s requirements, often involving a trade-off between simplicity and adaptability to complex spectral shapes. Experimentation with multiple algorithms is common to identify the most suitable approach for a given dataset. Explicit details can be found in the Supplementary Materials S1.

### 2.3. LSPR Biosensor Signal Acquisition through Visual Software

**Figure 1:**
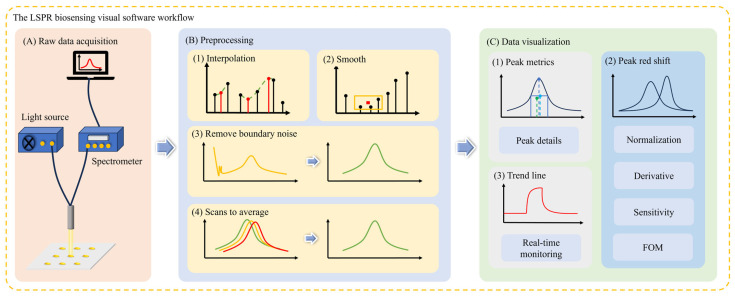
The LSPR biosensing visual software workflow. The workflow is divided into three parts: (**A**) raw spectra acquisition; (**B**) spectral data preprocessing; (**C**) result data visualization.

**Figure 2:**
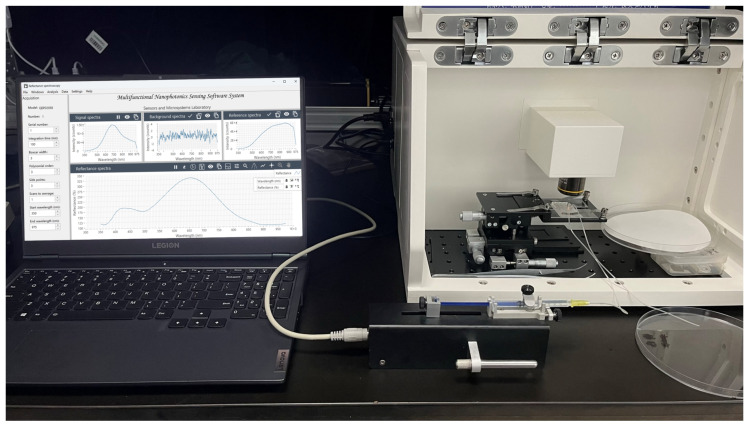
LSPR instrumentation and software system for biosensing monitoring.

To effectively utilize the raw data collected by the LSPR biosensor, a dedicated LSPR signal acquisition software was designed and developed to achieve efficient acquisition of spectral raw data, preprocessing of spectral data, and intuitive visualization of spectral data. After the raw data acquisition, the software performs preprocessing of the spectral data to improve the data quality. The LSPR biosensing visual software workflow is described in Figure 1, and the LSPR instrumentation and software system for biosensing monitoring are shown in Figure 2. This includes three parts: (1) Noise filtering, which uses advanced filtering algorithms to remove interfering signals from environmental interference or sensor noise. Explicit details can be found in the Supplementary Materials. (2) Signal calibration, which calibrates the spectral data according to the pre-set calibration standards to ensure the accuracy and comparability of the data. (3) Outlier handling, which detects and deals with possible outliers to prevent them from interfering with subsequent analysis. To enable the user to intuitively understand the output of the LSPR biosensors, the software provides powerful spectral data visualization capabilities. This involves the following: (1) real-time monitoring charts. Users could observe the changes in the LSPR spectral signal in real time to respond to the working status of the sensor in real time. (2) A historical trend graph provides historical trends of spectral signals to help users identify potential patterns and changes. (3) Spectra and peak analysis display the spectral map of the spectral data and analyze the peaks, enabling users to understand the sensor response more deeply.

**Figure 3:**
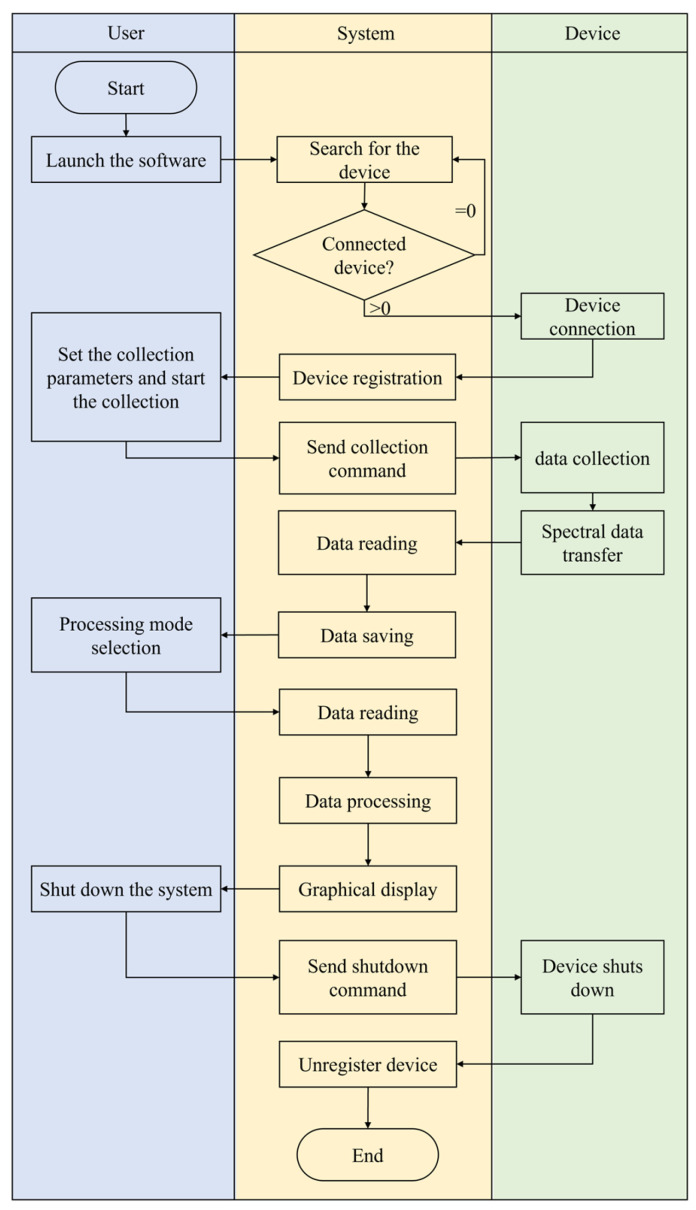
The LSPR biosensing integrated visual software flow chart.

To fulfill the integration requirements, the biosensing software system should possess the capability to guide users seamlessly throughout the entire process, starting from spectra generation and extending to processing. Specifically, the functions of this system can be divided into two main areas: device control and data processing. The former mainly involves setting the acquisition parameters of the sensor, completing the spectral acquisition, and receiving the transmitted data, while the latter aims to achieve comprehensive processing of the spectral data. This includes data processing and analysis according to user needs, performing operations such as smoothing [23], peak finding, baseline correction, spectral analysis, etc., to ensure the accuracy and reliability of the results. Such a comprehensive spectrometer software system will help to improve the efficiency and operability of spectroscopic experiments and meet the diverse needs of users in different fields. Combined with the specific needs of biosensing, the business process is summarized in Figure 3.

**Figure 4:**
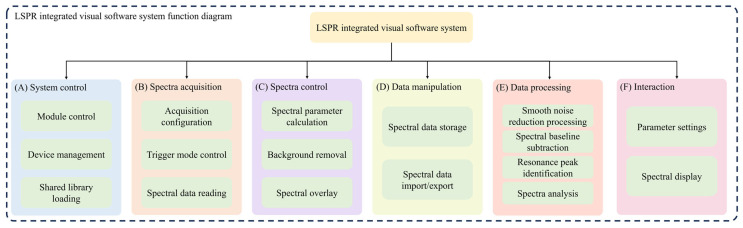
Functional diagram of the LSPR integrated visual software: (**A**) System control. The system controls multiple devices. (**B**) Spectra acquisition. The software system communicates with the sensors to collect and transmit spectra data. (**C**) Spectra control. The spectra collected from the sensor are processed. (**D**) Data manipulation. During the operation of the system, data access, file import, and export are implemented. (**E**) Data processing. The software system extracts features from the spectra and uses them for analysis. (**F**) interaction. The interactive interface provides human–computer interaction functions, such as database reading and writing, file operations, and beyond. This rigorous control extends to data access, as well as the import and export of files, guaranteeing seamless and efficient functionality.

The software system is mandated to address two primary requisites: equipment control and data processing. The software structure is shown in Figure 4. The main functions are system control, spectra acquisition, spectra control, analysis, and human–computer data and operation data processing interaction:

(1) System control. The software controls multiple devices and implements various algorithms for data processing. Therefore, in cases where multiple devices are involved, the system needs to manage the connected devices and choose from a variety of processing modes. In addition, the individual modules need to be managed and controlled.
(2) Spectra acquisition. According to the communication protocol, the software uses the serial port to transmit the control command to the sensor and realizes the communication with the sensor to collect and transmit spectral data.
(3) Spectra control. The spectra collected from the sensor are processed. According to the different needs of users, there will be different processing methods, mainly including the calculation of spectral characteristic parameters, background removal, spectral superposition, etc.
(4) Data manipulation. During system operation, data undergo meticulous management, encompassing tasks.
(5) Data processing. The system’s core functions revolve around data processing and analysis, encompassing advanced processing of acquired spectra, extraction of relevant features, and subsequent utilization of these features for thorough analysis. It reads data from the sensor, conducting calculations based on the user-selected mode to facilitate functions like noise reduction, spectral baseline subtraction, identification of feature peaks, and comprehensive spectral analysis.
(6) Interaction. The interactive interface acts as the channel for human–computer interaction and stands as the exclusive component of the system accessible to users. Consisting of a control interface and a spectra interface, this user interaction interface predominantly functions in observer mode, overseeing distinct controls and triggering diverse functions through an integration with the underlying model.

## 3. Visualization Implements of Integrated LSPR Signal Processing Software

An integrated processing and visualization strategy plays a pivotal role in enhancing the sensitivity of LSPR sensing. By seamlessly combining advanced data processing algorithms with sophisticated visualization techniques, this strategy enables the precise extraction and analysis of subtle signals captured by LSPR sensors. Through efficient processing, system noise is effectively mitigated, optimizing the signal-to-noise ratio and accentuating weak signals. The synergy of this integrated approach not only reduces the impact of noise but also provides researchers with a comprehensive and intuitive representation of complex sensing data. This holistic strategy empowers a deeper understanding of the intricate variations in LSPR sensing, ultimately contributing to heightened sensitivity. As a result, the integration of processing and visualization strategies not only refines the accuracy of LSPR sensors but also opens avenues for more nuanced and reliable sensing applications.

**Figure 5:**
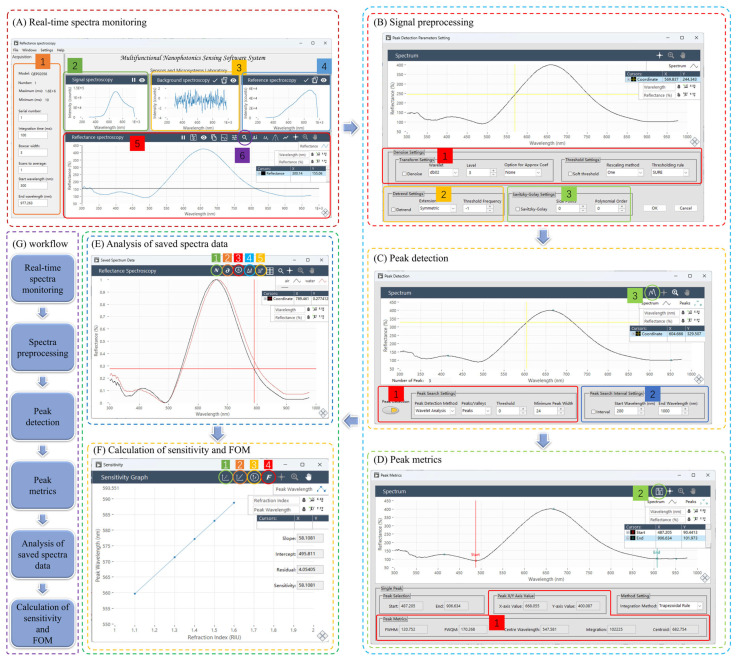
Software visualization implementation: (**A**) real-time spectra monitoring. (1) Acquisition parameters, which are used to preprocess the acquired raw spectral data to improve the accuracy of experimental results. (2) Signal spectra. (3) Backgroud spectra. (4) Reference spectra. (5) Reflectance spectra. (6) Peak detection. (**B**) Signal preprocessing. (1) Continuous wavelets transform denoise settings. (2) Baseline correction. (3) Savitzky-Golay filter settings. (**C**) Peak detection. (1) Settings of peak detection method, peak detection type, peak detection threshold, and minimum peak width. (2) Settings of the wavelength range applied to the peak detection algorithm. (3) Peak metrics. (**D**) Peak metrics. (1) Results of the resonance peak wavelength $\lambda_{max}$ and intensity $E_{max}$, FWHM, FWQM, center wavelength $\lambda_{c}$, peak area integration, and peak centroid $\lambda_{c}$. (2) Saving the resonance peak data. (**E**) Multiple spectra. The black curve in the figure E represents the reflectance spectrum of air and the red curve represents the reflectance spectrum of water. (1) Normalization of the spectral signal. (2) Derivative of the spectral signal. (3) Calculation of sensitivity. (4) Specifies a curve as the subtracted number and calculate the difference in intensity between multiple spectra. (5) The intensity difference between two specific spectral curves. (**F**) calculation of sensitivity and FOM. (1) Inserting data point of the refraction index and the resonance peak wavelength. (2) Linearization of the refraction index and the resonance peak wavelength and calculation of the sensitivity. (3) Converting the resonance peak wavelength into resonance peak energy to calculate the sensitivity in eV/RIU. (4) Calculation of FOM (figure of merit based on wavelength). (**G**) Workflow diagram for the visualization of the LSPR integrated software system.

The software system runs on the 64-bit Windows platform and is controlled by a graphical user interface; the system can detect absorption spectra, transmission spectra, reflection spectra, fluorescence spectra, Raman spectra, etc. Here, the reflectance spectra are taken as an example, and the software interface, such as Figure 5A, defines the initial parameters (1 in Figure 5A). Here, the indicator box “model” represents the model of the currently connected spectrometer, the indicator box “number” represents the total number of connected spectrometers, the indicator box “maximum” and “minimum” represent the integration time range of the currently connected spectrometer, the serial number represents the index of the spectrometer that is currently selected to read the data, and the averaging of scans represents the length of the continuous spectra calculation queue. Multiple spectra in a time series are averaged to eliminate the effects of random noise. The indicator boxes “start wavelength” and “end wavelength” specify the spectral data interval that the software system reads. This deliberate selection helps eliminate edge noise from the spectra. Following the configuration of the software system’s acquisition parameters, the next steps involve reading the signal (2 in Figure 5A), recording the background spectra (3 in Figure 5A), and capturing the reference spectra (4 in Figure 5A). These three key steps collectively contribute to the software’s automated calculation of the generated reflectance spectra (5 in Figure 5A). The software system contains automatic calculation modes such as absorption spectra, transmission spectra, reflectance spectra, Raman spectra, and fluorescence spectra. One system is capable of monitoring data between different types of spectrometers. After obtaining the reflectance spectra, load it into the peak finding preprocessing interface by clicking the peak finding button (6 in Figure 5A). Then, perform wavelet transform denoising (1 in Figure 5B) and baseline correction (2 in Figure 5B). After that, click the OK button to enter the software interface for peak detection (Figure 5C). Considering the sensitivity of the algorithm to wavelength movement and the real-time of the algorithm, the system uses a multi-scale wavelet transform to determine the position of the resonant wavelength. Users could set the method to be used to find the peak or valleys, the horizontal threshold, the interval of the minimum peak width (1 in Figure 5C), and the spectral wavelength range of the peak finding (2 in Figure 5C).

Click the peak metrics button (3 in Figure 5C) and enter the interface for characterization (Figure 5D) to calculate relevant sensing parameters for the characteristic peaks. In the characteristic peak characterization interface, drag and drop the start cursor and end cursor to set the wavelength range of the characteristic peak. The system automatically calculates the peak wavelength, peak intensity, centroid, center wavelength, integration, *FWHM*, and *FWQM* values (1 in Figure 5D). For the reflectance spectra detected under different refractive index conditions (*n_1_*, *n_2_*, *n_3_*), click the save button (2 in Figure 5D) to record the sensing parameters of the characteristic peaks of the reflectance spectra under each refractive index.

The stored spectral graph data are shown in Figure 5E. Using the button normalization (1 in Figure 5E) to normalize the spectral signal and using the derivative button (2 in Figure 5E), the relationship can be calculated between the first derivative of the spectra and the wavelength. Using the button ΔI (4 in Figure 5E) to calculate the ΔI of all other curves by using one curve as the subtracted number $I_{0}$, one curve could be chosen as $I_{1}$ and another curve as $I_{2}$ to calculate the difference in intensity $\Delta I_{i}=I_{i}-I_{0}$ between the two curves.

Sensitivity could be calculated using the sensitivity button (3 in Figure 5E). The software interface is as follows (Figure 5F): The insert data button (1 in Figure 5F). The sensitivity could be calculated by linear fitting (2 in Figure 5F) of the data in the graph. By using the button axis change (3 in Figure 5F), the resonance wavelength could be converted to eV units, and the sensitivity value could be calculated in eV/RIU units. Using the button (4 in Figure 5F), *FOM* could be calculated.

## 4. Results and Discussion

At the core of visual software lies a sophisticated array of signal-processing functions crucial for shaping and manipulating data in real time. The visual software is designed for the comprehensive processing of LSPR spectral signals, encompassing key LSPR sensor parameters such as resonance peak wavelength, amplitude, peak area, centroid, *FWHM*, *FWQM*, central wavelength, and the variation in these parameters with changes in refractive index. Additionally, the software includes crucial metrics like sensitivity, quality factor, spectral resolution, detection limit, signal-to-noise ratio, residuals, and coefficient of variation (*CV*). The transformative impact of signal processing on LSPR spectra becomes evident as we delve into a comparative analysis, unraveling the nuanced alterations and enhancements that transpire before and after the application of sophisticated signal processing techniques.

**Figure 6:**
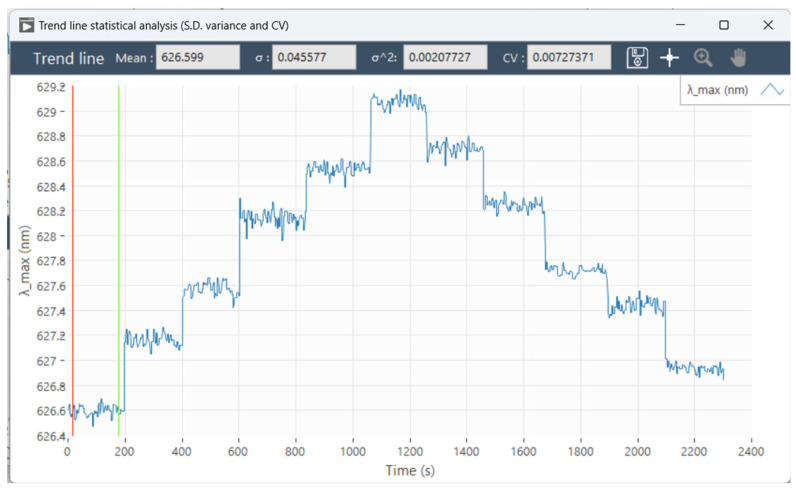
Analyzing the shift of resonance peak wavelength $\lambda_{max}$ time series data. The red and green lines are used to specify a range of intervals on the horizontal axis, which is used by the software system to calculate the mean, standard deviation, variance, and coefficient of variation of the signal data between these two lines in the graph.

**Figure 7:**
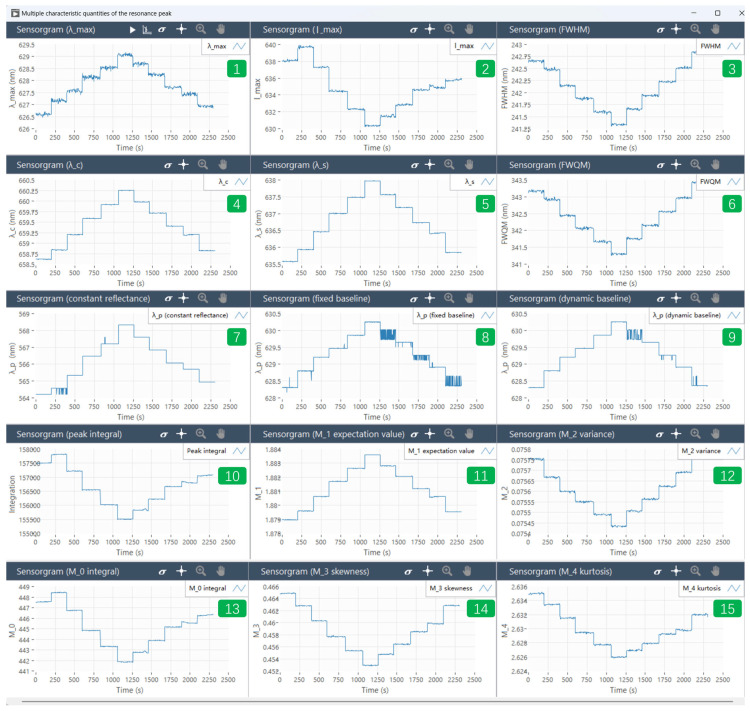
Multiple characteristic quantities of the resonance peak. The software system performs concurrent detection of various parameters: (**1**) resonance peak wavelength, (**2**) resonance peak amplitude, (**3**) full width at half maximum, (**4**) centroid wavelength, (**5**) center wavelength, (**6**) full width at quarter maximum, (**7**) resonance wavelength calculated by constant reflectance algorithm, (**8**) resonance wavelength calculated by fixed baseline algorithm, (**9**) resonance wavelength calculated by dynamic baseline algorithm, (**10**) integration of the resonance peak area, and (**11**–**15**) the quadruple central moments of the spectral distribution. The data for each time point are recorded in the software interface.

The reflectance spectra of 8 nm thermal evaporation rapidly annealed the formation of gold particles on LSPR sensing chips were detected in water, with 5%, 10%, 15%, 20%, and 25% NaCl in mass fractions. The mean value of the resonance peak wavelength characteristic quantities over time is taken as the data point in the linear fit to calculate the sensitivity. The standard deviation, variance, and coefficient of variation in the resonance peak shift were observed from the perspective of each resonance peak characterization quantity. When the standard deviation, variance, and coefficient of variation are low, the stability of the resonance wavelength of the characterization quantities is good. In Figure 6, the value of the resonance peak characterization is recorded for each moment of the spectra. Each flat line from left to right in the graph represents the value of the resonance peak characterization for one refractive index condition. A total of the graph contains the data of water and 5%, 10%, 15%, 20%, and 25% of the mass fraction of the NaCl solution, and the value of the resonance peak characterization for one refractive index condition was taken to calculate the standard deviation, square deviation, and coefficient of variation. The standard deviation, variance, and coefficient of variation were calculated to assess the accuracy of the resonance peak characterization or resonance peak wavelength calculation method. Specific data are given in Table 1. The software system can detect various parameters simultaneously, and Figure 7 shows the trend of multiple characteristic quantities of the resonance peaks as a function of time.

The data show that when monitoring the resonance peak through various characterization parameters, the resonance amplitude, center wavelength, and centroid wavelength display comparatively minor standard deviations and variances. This is because the centroid serves as the center of spectral distribution and is calculated through a wavelength-weighted average. The *FWHM* and central wavelength also show minimal deviation from the mean, indicating the stability of both the resonance center wavelength and the center of mass wavelength. However, the resonance area integral shows an elevated standard deviation, which is attributed to the absolute value of the resonance peak area integral. Parameters with lower coefficients of variation include the center of mass wavelength and center wavelength.

Moreover, when characterizing shifts in resonance peaks using fixed baseline, dynamic baseline, and fixed refractive index algorithms, their standard deviations, variances, and coefficients of variation are smaller in comparison to direct detection of resonance peak wavelengths. Despite the relatively large standard deviation in the peak area integral, the low coefficient of variation suggests that changes in peak area, although substantial, are not significant relative to the average.

**Figure 8:**
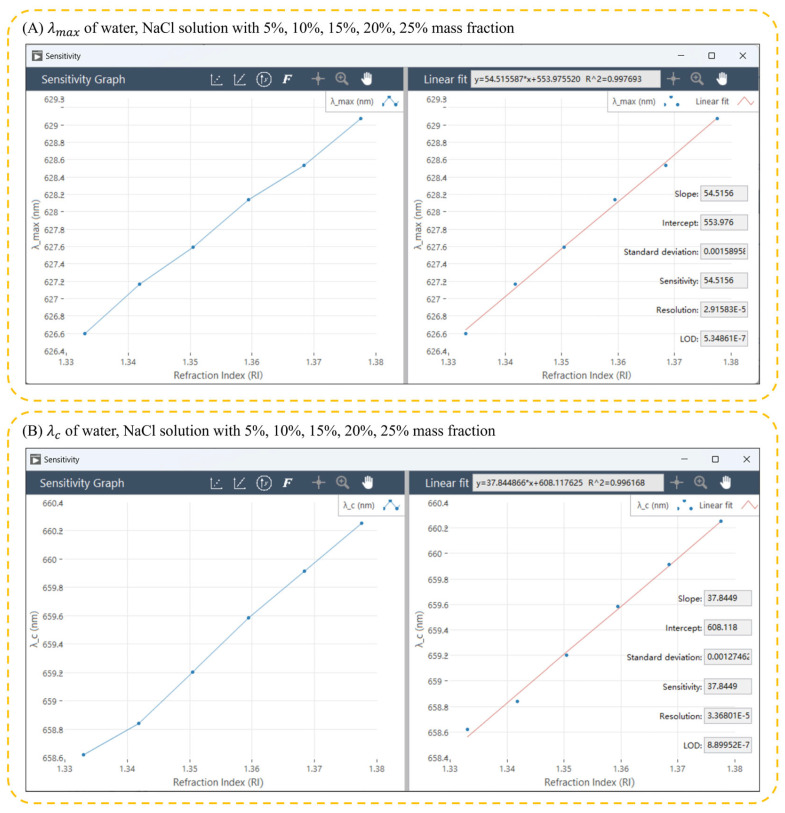
Sensitivity calculation. The reflectance spectra of Au nanoparticles were detected in water, with 5%, 10%, 15%, 20%, and 25% NaCl in mass fractions. The data in the figure is the resonance peak characteristic quantities (e.g., $\lambda_{max}$, $\lambda_{c}$, $\lambda_{s}$, *FWHM*, *FWQM*) under different refractive index solutions. The horizontal axis is the refractive index, and the vertical axis is the resonance peak characterization. The sensitivity of the sensor is calculated by linear fitting. (**A**) The value of $\lambda_{max}$ detected in water, NaCl solution with 5%, 10%, 15%, 20%, 25% mass fraction. (**B**) The value of $\lambda_{c}$ detected in water, NaCl solution with 5%, 10%, 15%, 20%, 25% mass fraction.

**Figure 9:**
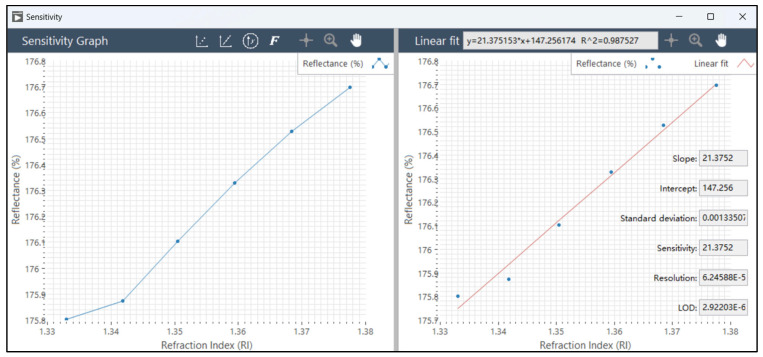
Sensitivity calculation. The reflectance spectra of Au nanoparticles were detected in water, with 5%, 10%, 15%, 20%, and 25% NaCl in mass fractions. The horizontal axis is the refractive index, and the vertical axis is the resonance peak intensity $I_{max}$.

To facilitate a comprehensive assessment of different resonance peak wavelength monitoring methods, the software system must consider sensitivity, figure of merit, resolution, and detection limit, thus enabling a thorough comparison of monitoring efficiency and data quality. Through a careful examination of these key performance indicators, we gain a nuanced understanding of the strengths and weaknesses of different methods in practical applications. This, in turn, provides a scientific foundation for selecting the most fitting monitoring solution tailored to a specific application scenario. As shown in Figure 8, the software system investigates the RI sensitivity, quality factor, resolution, and detection limit by recording the LSPR optical response when the LSPR sensing chip is immersed in different mass fractions of NaCl solutions. The results show that the three wavelength tracking algorithms have similar RI sensitivities (shown in Table 2), i.e., about 50 nm/RIU, for the algorithm that monitors the wavelength of the resonance peak, the fixed center-of-mass algorithm, and the dynamic center-of-mass algorithm. Moreover, the software system is capable of tracking variations in the intensity of the resonance peak in addition to its shift. The empirical outcomes of these observations are presented in Table 3 and illustrated in Figure 9. The algorithm that monitors the resonance peak wavelength by constant reflectance algorithm in this experiment has a much greater RI sensitivity than the other methods, with an RI sensitivity of 95.2644 nm/RIU. This increase in RI sensitivity can be attributed to the larger value of the change in the wavelength position at specific spectral intensities during the redshift of the peak of the LSPR. The method of monitoring the peak center of mass in the experiment has a small sensitivity, probably due to the smaller fluctuations in the peak center of mass and the smaller change value at the redshift of the LSPR peak.

When comparing the algorithms in terms of the figure of merit, the constant reflectance algorithm has the highest figure of merit, while the algorithm monitoring the *FWHM* has the lowest figure of merit. However, in terms of the standard deviation of the linear fit, the algorithm monitoring the *FWHM* has the lowest standard deviation. In contrast, the constant reflectance algorithm has the highest standard deviation of the linear fit. This result suggests that the algorithms may be sensitive to changes in the redshift of the LSPR peaks but, at the same time, may be weak to interference from noise. In practical application, the software system integrates different experimental conditions. Various commonly used algorithms for resonance peak displacement characterization are integrated to adapt to different experimental conditions.

Following the signal processing conducted by our software system, a meticulous analysis of LSPR spectral parameters is performed, comparing the values before and after processing. The software demonstrates a notable enhancement in LSPR sensor detection accuracy. This improvement is evident in the refined determination of resonance peak characteristics, leading to more accurate measurements of wavelength, amplitude, peak area, centroid, *FWHM*, *FWQM*, and central wavelength. Moreover, the software’s capacity to assess these parameters against variations in refractive index provides valuable insights into the sensitivity and responsiveness of the LSPR sensor under different conditions. Furthermore, the software elevates overall sensor performance by offering insights into additional factors critical for sensor evaluation, such as sensitivity, quality factor, spectral resolution, detection limit, signal-to-noise ratio, residuals, and coefficient of variation. This comprehensive approach not only enhances the precision of LSPR sensor measurements but also contributes to a deeper understanding of the sensor’s performance characteristics. In summary, our software system proves to be an indispensable tool in advancing the accuracy and reliability of LSPR sensor detection for a wide range of applications.

## 5. Conclusions

The integration of processing and visualization strategies has proven highly effective in advancing LSPR sensing technology. By seamlessly combining sophisticated data processing algorithms with intuitive visualization methods, we have not only elevated the sensitivity and accuracy of LSPR sensors but also deepened our understanding of sensing signals. This comprehensive approach has significantly contributed to the development of LSPR sensing technology, fostering its widespread applications in biomedical research, environmental monitoring, and beyond. Looking forward, future improvements should focus on refining integrated processing workflows for enhanced real-time adaptability, particularly in the face of complex environmental conditions and dynamic sample characteristics. Additionally, the evolution of visualization strategies should prioritize increased interactivity and user-friendliness to strengthen the interpretability of sensing data. As technology continues to evolve, LSPR sensing technology holds promising application prospects across various fields, including medical diagnostics, food safety, and beyond, showcasing its potential as a versatile and impactful sensing tool.

## Figures and Tables

**Table 1:** Standard deviations, variance, and coefficient of variation in the resonance wavelength calculated with different algorithms. Here, we are merely comparing the magnitude of numerical values; the specific dimensions can be derived from the corresponding calculation formulas in the Supplementary Materials.

Characteristic Parameters	Standard Derivation	Variance	Coefficient of Variation
$\lambda_{max}$	4.55770 × 10^−2^	2.07727 × 10^−3^	7.27371 × 10^−3^
$I_{max}$	6.28609 × 10^−2^	3.95149 × 10^−3^	9.85157 × 10^−3^
Peak area integral	4.85507	23.5717	3.08245 × 10^−3^
$\lambda_{c}$	3.77299 × 10^−3^	1.42355 × 10^−5^	5.72865 × 10^−4^
FWHM	1.52819 × 10^−2^	2.33537 × 10^−4^	6.29772 × 10^−3^
FWQM	2.10774 × 10^−2^	4.44257 × 10^−4^	6.14196 × 10^−3^
$\lambda_{s}$	5.04019 × 10^−3^	2.54035 × 10^−5^	7.93009 × 10^−4^
Constant reflectance	1.14081 × 10^−13^	1.30145 × 10^−26^	2.02198 × 10^−14^
Fixed baseline	2.55323 × 10^−3^	6.51897 × 10^−6^	4.06364 × 10^−4^
Dynamic baseline	1.50999 × 10^−3^	2.28008 × 10^−6^	2.40326 × 10^−4^
$M_{0}$ Integral	1.74456 × 10^−2^	3.04349 × 10^−4^	3.89805 × 10^−3^
$M_{1}$ Expectation value	8.78245 × 10^−6^	7.71315 × 10^−11^	4.67409 × 10^−4^
$M_{2}$ Variance	2.91788 × 10^−6^	8.51404 × 10^−12^	3.85185 × 10^−3^
$M_{3}$ Skewness	3.50356 × 10^−5^	1.22749 × 10^−9^	7.53655 × 10^−3^
$M_{4}$ Kurtosis	3.49702 × 10^−5^	1.22291 × 10^−9^	1.32713 × 10^−3^

**Table 2:** Sensitivity (*S*, nm/RIU), figure of merit (*FOM*), standard deviation of linear fit of spectral resonance peak shift (*σ*), spectral resolution (*R*) and limit of detection (*LOD*) calculated by the spectral resonance peak shift monitoring data through characteristic quantities.

Characteristic Parameters	*S* (nm/RIU)	*FOM* (RIU^−1^)	σ (Linear Fit, nm)	*R* (RIU)	*LOD* (RIU^2^/nm)
$\lambda_{max}$	54.5156	0.224660	1.58958 × 10^−3^	2.91583 × 10^−5^	5.34861 × 10^−7^
$\lambda_{c}$	37.8449	0.155959	1.27462 × 10^−3^	3.36801 × 10^−5^	8.89952 × 10^−7^
FWHM	30.5045	0.125709	7.68332 × 10^−4^	2.51875 × 10^−5^	8.25696 × 10^−7^
FWQM	43.1601	0.177863	1.56704 × 10^−3^	3.63075 × 10^−5^	8.41229 × 10^−7^
$\lambda_{s}$	55.0763	0.226970	1.68602 × 10^−3^	3.06124 × 10^−5^	5.55818 × 10^−7^
Fixed baseline	42.1106	0.173538	2.63576 × 10^−3^	6.25914 × 10^−5^	1.48636 × 10^−6^
Dynamic baseline	42.1075	0.173526	2.64558 × 10^−3^	6.28291 × 10^−5^	1.49211 × 10^−6^
Constant reflection	95.2644	0.392587	3.55440 × 10^−2^	3.73109 × 10^−4^	3.91657 × 10^−6^

**Table 3:** Sensitivity (*S*, A/RIU), figure of merit (*FOM*), standard deviation of linear fit of spectral resonance peak shift (*σ*), spectral resolution (*R*), and limit of detection (*LOD*) calculated by the spectral resonance peak intensity monitoring data. Here, the unit A means reflectance (%).

**Characteristic Parameters**	***S* (A/RIU)**	***FOM* (RIU** **^−1^)**	σ ** (Linear Fit, A)**	***R* (RIU)**	***LOD* (RIU^2^/A)**
$I_{max}$	21.3569	8.80116 × 10^−2^	1.33507 × 10^−2^	6.24588 × 10^−5^	2.92203 × 10^−5^

## Data Availability

The raw data supporting the conclusions of this article will be made available by the authors upon request.

## Supplementary Materials

The following supporting information can be downloaded at: https://www.mdpi.com/article/10.3390/mi15050631/s1, Figure S1: The algorithms for determining LSPR resonance peak wavelength [24,25]; Figure S2: LSPR sensing parameters [26]; Figure S3: SNR calculation; Figure S4: Interpolation method integrated on the integrated visual software [27,28,29,30]; Figure S5: Smoothing algorithms integrated on the visual software; Table S1: The workflow of multi-scale wavelet-continuous peak detection.; Table S2: Summary of the algorithm for determining LSPR resonance peak wavelength; Table S3: Summary of the LSPR sensing parameters integrated into the visual software; Table S4: Summary of preprocessing methods integrated into the visual software [31,32,33,34,35,36,37]; Table S5: The workflow of baseline correction.

## References

[1] He J., Wu S., Chen W., Kim A., Yang W., Wang C., Gu Z., Shen J., Dai S., Chen W. (2023). Calligraphy of Nanoplasmonic Bioink-Based Multiplex Immunosensor for Precision Immune Monitoring and Modulation. ACS Appl. Mater. Interfaces.

[2] Xu T., Geng Z. (2021). Strategies to Improve Performances of LSPR Biosensing: Structure, Materials, and Interface Modification. Biosens. Bioelectron..

[3] Proença M., Rodrigues M.S., Borges J., Vaz F. (2020). Optimization of Au:CuO Nanocomposite Thin Films for Gas Sensing with High-Resolution Localized Surface Plasmon Resonance Spectroscopy. Anal. Chem..

[4] Palani S., Kenison J.P., Sabuncu S., Huang T., Civitci F., Esener S., Nan X. (2023). Multispectral Localized Surface Plasmon Resonance (msLSPR) Reveals and Overcomes Spectral and Sensing Heterogeneities of Single Gold Nanoparticles. ACS Nano.

[5] Huang T., Wang J., Duan F., Jiang J., Fu X., Xu X. (2019). A Simple Algorithm for the Implementation of Second-Order-Polynomial Based Peak-Tracking Methods. Opt. Fiber Technol..

[6] Yang G., Dai J., Liu X., Chen M., Wu X. (2020). Spectral Feature Extraction Based on Continuous Wavelet Transform and Image Segmentation for Peak Detection. Anal. Methods.

[7] Gul M.U., Kadir K., Azman H.K., Iqbal S. Detection of R-Peaks Using Single-Scale Wavelet Transform. Proceedings of the 2019 13th International Conference on Mathematics, Actuarial Science, Computer Science and Statistics (MACS).

[8] Ma K., Liu L., Zhang P., He Y., Peng Q. (2019). Optimization of Angle-Pixel Resolution for Angular Plasmonic Biosensors. Sens. Actuators B Chem..

[9] Wang G., Shi J., Zhang Q., Wang R., Huang L. (2022). Resolution Enhancement of Angular Plasmonic Biochemical Sensors via Optimizing Centroid Algorithm. Chemom. Intell. Lab. Syst..

[10] Jeon J., Uthaman S., Lee J., Hwang H., Kim G., Yoo P.J., Hammock B.D., Kim C.S., Park Y.-S., Park I.-K. (2018). In-Direct Localized Surface Plasmon Resonance (LSPR)-Based Nanosensors for Highly Sensitive and Rapid Detection of Cortisol. Sens. Actuators B Chem..

[11] Baihaqi M.Y., Lumoindong C.W.D., Vincent V. (2021). Simulasi Perbandingan Filter Savitzky Golay Dan Filter Low Pass Butterworth Pada Orde Ketiga Sebagai Pembatal Kebisingan. KONSTELASI Konvergensi Teknol. Sist. Inf..

[12] Zhang G., Hao H., Wang Y., Jiang Y., Shi J., Yu J., Cui X., Li J., Zhou S., Yu B. (2021). Optimized Adaptive Savitzky-Golay Filtering Algorithm Based on Deep Learning Network for Absorption Spectroscopy. Spectrochim. Acta Part A.

[13] Zhang X., Liu Z., Wang J., Wang J. (2019). Time–Frequency Analysis for Bearing Fault Diagnosis Using Multiple Q-Factor Gabor Wavelets. ISA Trans..

[14] Altenhof A.R., Mason H., Schurko R.W. (2023). DESPERATE: A Python Library for Processing and Denoising NMR Spectra. J. Magn. Reson..

[15] Zhang X., Qi W., Cen Y., Lin H., Wang N. (2019). Denoising Vegetation Spectra by Combining Mathematical-Morphology and Wavelet-Transform-Based Filters. J. Appl. Remote Sens..

[16] Krämer S.D., Wöhrle J., Rath C., Roth G. (2019). Anabel: An Online Tool for the Real-Time Kinetic Analysis of Binding Events. Bioinf. Biol. Insights.

[17] Costa E.B., Rodrigues E.P., Pereira H.A. (2019). Sim-SPR: An Open-Source Surface Plasmon Resonance Simulator for Academic and Industrial Purposes. Plasmonics.

[18] Dahl G., Steigele S., Hillertz P., Tigerström A., Egnéus A., Mehrle A., Ginkel M., Edfeldt F., Holdgate G., O’Connell N. (2017). Unified Software Solution for Efficient SPR Data Analysis in Drug Research. SLAS Discov..

[19] Rodrigues M.S., Pereira R.M.S., Vasilevskiy M.I., Borges J., Vaz F. (2020). NANOPTICS: In-Depth Analysis of NANomaterials for OPTICal Localized Surface Plasmon Resonance Sensing. SoftwareX.

[20] Muri H.I., Bano A., Hjelme D.R. (2018). A Single-Point, Multiparameter, Fiber Optic Sensor Based on a Combination of Interferometry and LSPR. J. Light. Technol..

[21] Paul D., Biswas R. (2018). Highly Sensitive LSPR Based Photonic Crystal Fiber Sensor with Embodiment of Nanospheres in Different Material Domain. Opt. Laser Technol..

[22] Santos C., Dias C. (2021). Note on the Coefficient of Variation Properties. Braz. Electron. J. Math..

[23] Çataltaş Ö., Tutuncu K. (2021). A Review of Data Analysis Techniques Used in Near-Infrared Spectroscopy. Eur. J. Sci. Technol..

[24] Chinowsky T.M., Jung L.S., Yee S.S. (1999). Optimal Linear Data Analysis for Surface Plasmon Resonance Biosensors. Sens. Actuators B Chem..

[25] Thirstrup C., Zong W. (2005). Data Analysis for Surface Plasmon Resonance Sensors Using Dynamic Baseline Algorithm. Sens. Actuators B Chem..

[26] Dos Santos P.S.S., Mendes J.P., Dias B., Pérez-Juste J., De Almeida J.M.M.M., Pastoriza-Santos I., Coelho L.C.C. (2023). Spectral Analysis Methods for Improved Resolution and Sensitivity: Enhancing SPR and LSPR Optical Fiber Sensing. Sensors.

[27] Shao L., Lin X., Shao X. (2002). A Wavelet Transform and Its Application to Spectroscopic Analysis. Appl. Spectrosc. Rev..

[28] Savitzky A., Golay M.J.E. (1964). Smoothing and Differentiation of Data by Simplified Least Squares Procedures. Anal. Chem..

[29] Milojković-Opsenica D., Ristivojević P., Andrić F., Trifković J. (2013). Planar Chromatographic Systems in Pattern Recognition and Fingerprint Analysis. Chromatographia.

[30] Leung A.K., Chau F., Gao J. (1998). A Review on Applications of Wavelet Transform Techniques in Chemical Analysis: 1989–1997. Chemom. Intell. Lab. Syst..

[31] Kwiatkowski A., Gnyba M., Smulko J., Wierzba P. (2010). Algorithms of Chemicals Detection Using Raman Spectra. Metrol. Meas. Syst..

[32] Zhang F., Tang X., Tong A., Wang B., Wang J. (2020). An Automatic Baseline Correction Method Based on the Penalized Least Squares Method. Sensors.

[33] Ruckstuhl A.F., Jacobson M.P., Field R.W., Dodd J.A. (2001). Baseline Subtraction Using Robust Local Regression Estimation. J. Quant. Spectrosc. Radiat. Transfer.

[34] Jiang X.Q., Blunt L., Stout K.J. (2000). Development of a Lifting Wavelet Representation for Surface Characterization. Proc. R. Soc. Lond. A Math. Phys. Eng. Sci..

[35] Hoang V.D. (2014). Wavelet-Based Spectral Analysis. TrAC Trends Anal. Chem..

[36] Perez-Pueyo R., Soneira M.J., Ruiz-Moreno S. (2010). Morphology-Based Automated Baseline Removal for Raman Spectra of Artistic Pigments. Appl. Spectrosc..

[37] Zhang Z.-M., Chen S., Liang Y.-Z., Liu Z.-X., Zhang Q.-M., Ding L.-X., Ye F., Zhou H. (2010). An Intelligent Background-Correction Algorithm for Highly Fluorescent Samples in Raman Spectroscopy: Background-Correction Algorithm for Highly Fluorescent Samples in Raman Spectroscopy. J. Raman Spectrosc..

