# Supplementary material for: Integrated LSPR Biosensing Signal Processing Strategy and Visualization Implementation
Source: Micromachines (Basel). 2024 May 8;15(5):631. doi: 10.3390/mi15050631 (PMC11123047; doi:10.3390/mi15050631)
Supplement: Supplementary file 1 [file micromachines-15-00631-s001.zip › micromachines-2969198-supplementary.pdf]

Supplementary Materials

Integrated LSPR biosensing signal processing strategy and visualization implementation

Mixing Zhou [1] and Zhaoxin Geng [1,2*1] School of Information Engineering, Minzu University of China, Beijing 100081, China; 21301983@muc.edu.cn (M. Z.); zxgeng@muc.edu.cn (Z. G.)
[2] Key Laboratory of Ethnic Language Intelligent Analysis and Security Governance of MOE, Minzu University of China, Beijing 100081, China; zxgeng@muc.edu.cn
* Correspondence: zxgeng@muc.edu.cn

S1. Multiple resonance peak characterization algorithms

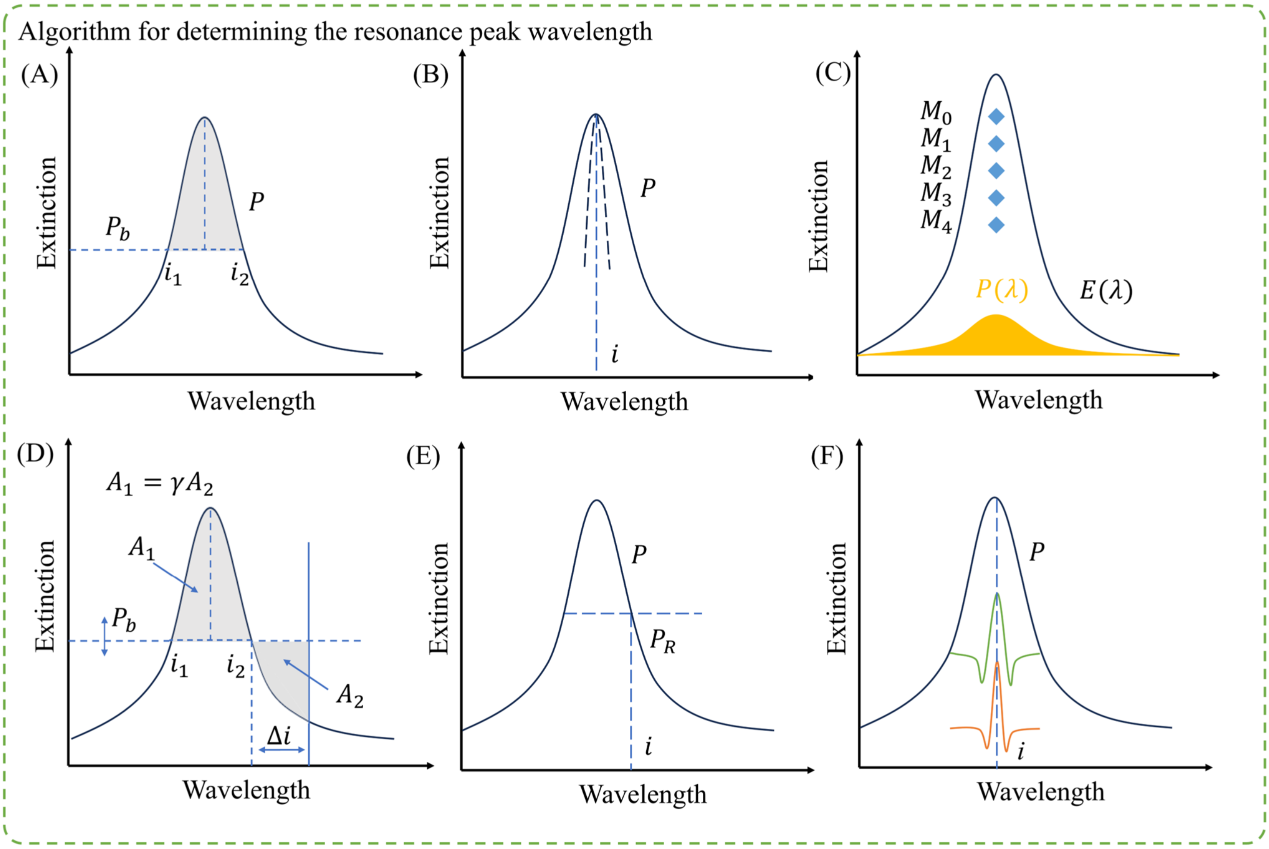

Figure S1. The algorithms for determining LSPR resonance peak wavelength. (A) Centroid algorithm with a fixed baseline. (B) Polynomial fit algorithm. (C) Quadruple central moments algorithm. (D) Dynamic baseline algorithm. (E) Constant reflectance algorithm. (F) Multi-scale continuous wavelet transform algorithm.

Fig. S1 (A) represents the centroid algorithm with a fixed baseline [1]. This equation defines the algorithm known as the first-order moment or center of gravity: This equation applies only to wavelength i where the difference between the LSPR curve P and the fixed baseline P_0 is positive.

$$\lambda_{max} = \frac{\sum_{i=i_1}^{i_2}(P_{i,b} - P_i)i}{\sum_{i=i_1}^{i_2}(P_{i,b} - P_i)} \tag{1}$$

The fixed baseline P_0 remains constant for all wavelengths. The formula gives a minimum value of P to which the resonance point (non-integer) does not normally correspond; the formula indicates that the light intensity fluctuation will affect the value of the calculated resonance point because the baseline remains the same while the scale α/β pressed due to the fluctuation. In addition, the choice of a fixed baseline affects the value of i_1 and i_2 in the formula and could significantly change the value of the resonance point, even for small fluctuations in light intensity, which could be avoided by using a dynamic baseline algorithm due to the asymmetrical inclination in the LSPR curve. The outstanding advantage of this method is that it is simple and fast. The disadvantage of this method is sensitive to fluctuations in light intensity.

Fig. S1 (B) represents the polynomial fitting algorithm [2]. The polynomial fitting algorithm is executed at the position where the LSPR curve is minimum. The fourth-order polynomial equation was used to fit the asymmetric LSPR spectral curve to find its minimum value. The fourth-order polynomial equation is fitted to 10 points on either side of the minimum wavelength in the LSPR spectrum. The resonance point is determined by locating the minimum value of the fitted polynomial. This method is not affected by fluctuations in light intensity, as α/β scaling the LSPR spectral distribution does not change the position of its minimum.

Fig. S1 (C) represents the quadruple central moments algorithm [3]. The statistical analysis of LSPR spectra with normalized spectral distributions was performed to compare different sensing platforms in this method, to compute the four statistical central moments of the spectral distributions, we employ a normalization process on both the wavelength and amplitude, resulting in the derivation of the distribution function *P(λ)*. Subsequently, the central moments of *P(λ)* are determined: the first moment (M_1, the expectation value), the second moment (M_2, the variance), and the third moment (M_3, skewness), and the fourth moment (M_4, kurtosis).

$$\lambda = \frac{\lambda'}{\lambda_{min}} \tag{2}$$

Where, λ is normalized wavelength; λ' is the wavelength of original spectra; λ_{min} is the minimum of the wavelength range of the original spectra.

$$M_0 = \int_{L_1}^{L_2} E(\lambda)\, \mathrm{d}\lambda \tag{3}$$

Where M_0 is the peak area integration between wavelengths L_1 and L_2; $E(\lambda)$ is the amplitude of the spectrum at wavelength λ.

$$P(\lambda) = \frac{E(\lambda)}{M_0} \tag{4}$$

$$M_1 = \int_{L_1}^{L_2} \lambda P(\lambda)\, \mathrm{d}\lambda \tag{5}$$

$$M_2 = \int_{L_1}^{L_2} (\lambda - M_1)^2 P(\lambda)\, \mathrm{d}\lambda \tag{6}$$

$$M_3 = \frac{\int_{L_1}^{L_2} (\lambda - M_1)^3 P(\lambda)\, \mathrm{d}\lambda}{M_2^{\frac{3}{2}}} \tag{7}$$

$$M_4 = \frac{\int_{L_1}^{L_2} (\lambda - M_1)^4 P(\lambda)\, \mathrm{d}\lambda}{{M_2}^2} \tag{8}$$

Where $P(\lambda)$ is the normalized $E(\lambda)$; M_1 is the first moment (Expectation value); M_2 is the second moment (the variance); M_3 is the third moment (skewness) and M_4 is the fourth moment (kurtosis).

Fig. S1 (D) represents the dynamic baseline algorithm [4]. Because the baseline P_b is defined dynamically, the ratio of the area above and below the baseline is constant even after the introduction of light intensity fluctuations. The region A_1 above the baseline is acquired between the lower limit of the wavelength defined by the constant shift Δi of the wavelength that is minimal relative to the LSPR spectrum, and the wavelength at which the baseline intersects with the LSPR spectral profile. A_2 locates between the first wavelength and the second wavelength where the baseline intersects the LSPR spectrum, the area of these two regions is calculated using the trapezoidal method. To maintain the same area ratio of the newly measured LSPR spectrum as the newly measured LSPR spectrum, the baseline needs to be redefined. Once a new baseline has been determined, the resonant point wavelength is calculated using the centroid algorithm, which reduces the effect of light intensity fluctuations.

Fig. S1 (E) represents the constant reflectance algorithm [5]. This simple method lies in finding the wavelength that corresponds to the preselected values P_R. On the right side of the LSPR spectrum, select the constant $0.8P_R$ value. The quadratic polynomial is fitted to 10 points on either side of a wavelength of about $0.8Pr$ in the LSPR spectrum to determine the wavelength at resonance. As with the centroid algorithm, the algorithm is susceptible to fluctuations in light intensity when introduced, as the LSPR spectral distribution scales as α/β according to the fluctuations.

Fig. S1 (F) represents the multi-scale continuous wavelet transform algorithm [6], which is mainly divided into six steps, which are data interpolation, wavelet transform, ridge acquisition, ridge correction, ridge screening, and peak position correction. After the spectral data is interpolated, the continuous wavelet transform is performed on the spectral data, the wavelet coefficients at different scales are calculated, the ridges are obtained from the wavelet coefficient matrix, each ridge is corrected, the offset degree of the ridge is corrected, each ridge is screened by using the ridge length, approximate peak width and signal-to-noise ratio, and the peak position obtained by each ridge is merged and corrected by using the sliding window.

The continuous wavelet transform is used to achieve pattern matching. This algorithm adds the calculation of the continuous spectrum and uses the time characteristics of the spectrum to eliminate random noise. In addition, due to the limitation of the parent small crest type, the spectral peaks of special peak types may not be identified, and the characteristic peaks of this type could be identified through continuous spectrum calculation, which could improve the accuracy of peak finding.

The pseudocode is shown in Table S1. Each step in the process is described in it.

Table S1. The workflow of multiscale wavelet-continuous peak detection.

Algorithm 2 multiscale wavelet-continuous peak detection
Input: $S = \{(x_1, y_1), (x_2, y_2), \mathrm{I}.\,(x_n, y_n)\}$, scale, wavelet

```
Output: Peaks results P
Calculate interpolation Gap Δs;
S = Interpolation(S, Δs);
Calculate cwt coefficient matrix C(S, scale, wavelet);
for i = size(C, 2): 1
    Get maxima array M_i
    if i == size(C, 2): Initialize searchlist(M_i); Set Gap(M_i);
for M_i in searchlist
    if Gap(M_i)>threshold: Remove searchlist(M_i)
Get ridge matrix R;
for R_i in R: Correct ridge R_i;
for R_i in R
    Get parameters SNR_i, rl_i, rW_i;
    Filter by SNR_i, rl_i, rW_i;
    Update ridge matrix R;
Get peaks array P;
for P_i in P
    Get current scale a;
    Set window size ws = a;
    while ws <4*a:
        if P_i is real peak: continue;
        ws =2*ws;
    delete P_i
Return P
```

- **Procedure for the multiscale wavelet-continuous peak detection**:

1. The spectral data is interpolated so that the wavelength is normalized. Considering that the interpolation result will affect the operation efficiency of the algorithm, the interpolation interval is set to half of the minimum wavelength interval for simple spectra and the minimum wavelength interval for complex spectra.
2. The continuous wavelet transform was performed on the spectral data to obtain the wavelet coefficients $C = \{C_0, C_1, \dots\dots, C_n\}$ at different scales.
3. To get the ridges from the wavelet coefficient matrix C. (a) set $i = N$, get the maximum array of the wavelet coefficient array C_i, use the maximum array to initialize the search list $searchlist$, and set the interval value $gap = 0$ for each ridge. (b) Set $i = i - 1$, get the maximum array of wavelet coefficients C_i. For each ridge in $searchlist$, identify the closest peak and link them together, and set the interval $gap = 0$. If it is not found, set the interval $gap = gap + 1$ for the ridge. (c) For maximums that are not connected, add them to the maximum points of the $searchlist$ and set the interval $gap = 0$. (d) Each ridge in the $searchlist$ is judged, and if its interval gap is greater than the threshold value, it is removed from $searchlist$ and saved to the result list. (e) Repeat steps (b), (c), and (d) until the wavelet coefficient matrix is traversed.
4. This process corrects the degree of offset in each ridge. For each ridge line, starting from the largest scale $a = a_{max}$, in order from large to small, according to Equation 3, the point is eliminated if the condition is satisfied, and if it is not satisfied, the ridge correction is completed.
5. Ridge lines are filtered. Using the three indicators of ridge length, approximate peak width, and signal-to-noise ratio. (a) calculate the signal-to-noise ratio for each ridge in the ridge array $ridge_0$, as shown in the following formula:

$$SNR = \frac{C_{max}}{0.95 \sum_{i=1}^{n} C_i} \tag{9}$$

Where C_{max} is the maximum wavelet coefficient on the ridge and C_i is the wavelet coefficient at the scale $a = a_0$, and the signal-to-noise ratio array is obtained. For the SNR array calculated by the above formula, the Generalized Extreme Studentized Deviate (GESD) algorithm is used to obtain the outliers and remove the outliers. This method is a statistical-based outlier detection method, and its evaluation index is extreme student residuals R_i. The following describes how to calculate the set $\{x_1, ..., x_n\}$ of length n.

$$R_i = \frac{x_i - \bar{x}}{s} \tag{10}$$

Where s is the standard deviation, $\bar{x}$ is the mean value of the data. According to the above formula, the outliers are judged by comparing R_i with the critical value, λ_i in each calculation.

$$\lambda_i = \frac{(n-1)t_{n-i-1,p}}{\sqrt{(n-i-1)\left(n-i-1+{t_{n-i-1,p}}^2\right)}} \tag{11}$$

Where $t_{n-i-1,p}$ is the value when the probability of the right tail of the t-distribution with degrees of freedom $n-i-1$ is p. The p is calculated as follows:

$$p = \frac{\alpha}{2(n-i-1)} \tag{12}$$

α is the probability of an error that is allowed.

After that, the average signal-to-noise ratio $\overline{SNR}$ is calculated for the remaining data of the array, and the threshold is set as $1.1\overline{SNR}$ to filter the ridges to obtain the target ridge array $ridge_1$. (b) The ridge length rl_i is calculated for each ridge in the ridge array $ridge_0$, which is defined as the number of points on the ridge line. Based on the length of the ridge, the array $ridge_0$ is calculated by using the GESD algorithm to obtain an outlier array $ridge_2$. (c) Calculate the approximate peak width rw_i for each ridge in the ridge array $ridge_0$ by the scale corresponding to the maximum amplitude on the ridge, i.e.$a_{max}w = 2a_{max}$. Based on the length of the ridge, the array $ridge_0$ is calculated by using the GESD algorithm to obtain an outlier array $ridge_3$. (d) Find the intersection U_1 of array $ridge_1$ and array $ridge_2$, the intersection U_2 of array $ridge_1$ and array $ridge_3$ and then find the union of array U_1 and array U_2, which is the filtered ridge set.

6. Using the sliding window to correct and merge the peaks P obtained for each ridge. (a) The initial window half-width is set to $ws = a_{max}$, The window range is $[P, -ws, P_{i+ws}]$. (b) Looking for the maximum point in the range within the window, if this maximum is an outlier found by the GESD method and is not the first or last peak in the window, then the position of this point is the corrected peak, otherwise, proceed to step (c). (c) Doubles the half-width of the window ws.If the $ws > 4a_{max}$ window, then delete the ridge, otherwise, go to Step (b).

Table S2. Summary of the algorithm for determining LSPR resonance peak wavelength.

Name	Advantages	Disadvantages
Centroid algorithm with a fixed baseline	Simple and quick.	Sensitive to fluctuations in light intensity.
Polynomial fitting algorithm	Simple and flexible.	Overfitting, sensitivity to outliers, extrapolation issues, and choice of degree.
Dynamic baseline algorithm	Adaptability, real-time adjustment, and improved accuracy.	Complexity.
Constant reflectance algorithm	Simple and stable.	Limited applicability and inaccuracy in varied conditions.
Multi-scale continuous Wavelet transform algorithm	Multiresolution analysis, flexibility in scale selection, and feature extraction.	Computational complexity, selection of wavelet function, and boundary effects.
Quadruple central moments algorithm	The normalized spectral calculation central moments are suitable for comparison of various types of spectral data.	The calculation steps are complex

S2. Multiple characteristic quantities in observing the red shift of resonance peak

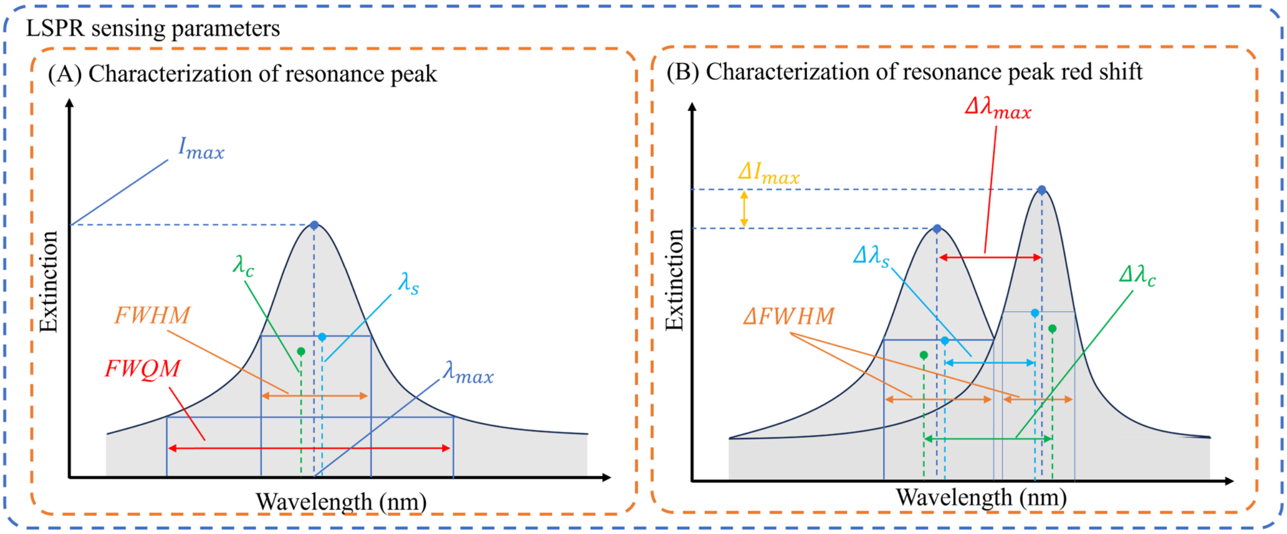

Figure S2. LSPR sensing parameters. (A) Relevant sensing parameters used to observe the center position of the resonance peak. (2) Relevant sensing parameters used to observe changes in the central position of resonance peaks.

The calculation formulas are expressed as follows:

$$\Delta FWHM = FWHM' - FWHM \tag{1}$$

$$\Delta FWQM = FWQM' - FWQM \tag{2}$$

$$\Delta\lambda_c = \lambda_c' - \lambda_c \tag{3}$$

$$\Delta\lambda_s = \lambda_s' - \lambda_s \tag{4}$$

Where $FWHM'$; $FWQM'$; λ_c' and λ_s'means the value after reaction; *FWHM*; *FWQM*; λ_c and λ_s means the value before reaction.

Sensitivity in sensing refers to the ability of LSPR sensing chip to detect small changes or variations in the quantity when measuring. For LSPR biosensors, sensitivity indicates how effectively the sensor could respond to minute changes in the refractive index of the surrounding medium.

$$S = \frac{d\lambda_p}{dn} \tag{5}$$

Where S denotes the sensitivity; n is the refractive index and λ_p is the resonance peak wavelength. Larger nanoparticles typically demonstrate increased sensitivities, although their peaks may be broadened due to multipolar excitations and radiative damping. The figure of merit (*FOM*), extensively utilized to assess a nanoparticle's sensing capabilities, is calculated by dividing the sensitivity by the resonance line width.

$$FOM = \frac{S}{FWHM} \tag{6}$$

Where S is the sensitivity; *FWHM* is the full width at half maximum. Measure the variation of the sensor peak at different refractive indexes, perform a linear fit for the displacement of the refractive index and the peak, calculate the standard deviation, Resolution (R) was then calculated by dividing this standard deviation (σ) by the RI sensitivity (S) as [7]

$$R = \frac{\sigma}{S} \tag{7}$$

Where R is the spectral resolution; $\Delta\lambda_{min}$ means the minimum value that could be measured by LSPR biosensors, which refers to the capability of a spectrometer to differentiate between different wavelengths during the measurement of a spectrum [8]. In optical sensing, higher spectral resolution allows for finer discrimination between wavelengths, providing more detailed information about the target or environment being measured. Improved spectral resolution is particularly valuable in applications where precise identification of spectral features is crucial.

The limit of detection (*LOD*) is a critical parameter that defines the smallest amount or concentration of a substance that a sensor could reliably detect. It serves as a measure of the sensor capability to discern low levels of analytes in a sample. *LOD* is defined as follows [8]:

$$LOD = \frac{R}{S} \tag{8}$$

Where R means the spectral resolution; S means the sensitivity. A lower limit of detection values indicates higher sensitivity and is essential for applications where trace amounts of substances need to be identified or monitored, such as in medical diagnostics and environmental sensing. The various LSPR sensing parameters integrated into the software system are summarized in Table S3.

Table S3. Summary of the LSPR sensing parameters integrated on the visual software.

Parameters	Definition	Signification
λ_{max}	Resonance peak wavelength	Molecular binding events, analyte concentration, real-time monitoring
I_{max}	Resonance peak intensity	Quantitative analysis, Signal-to-Noise ratio
$Integration$	Resonance peak area	Quantitative analysis and qualitative analysis
λ_c	Resonance peak centroid	Average resonance position, quantitative analysis, sensitivity enhancement, reducing noise effects
$FWHM$	The width of a line shape at half of its maximum amplitude	Sharpness of resonance peak, spectral resolution, discrimination of multiple peaks, reducing noise effects, quality of plasmon resonance
$FWQM$	The width of a line shape at a quarter of its maximum amplitude	Sharpness of resonance peak, spectral resolution, discrimination of multiple peaks, reducing noise effects, quality of plasmon resonance
λ_s	The center of the FWHM	Average resonance position, quantitative analysis, sensitivity enhancement, reducing noise effects
$\Delta\lambda_{max}$	The shifting of resonance peak wavelength	Monitoring molecular binding events
ΔI_{max}	The change of resonance peak amplitude	Monitoring molecular binding events
$\Delta\lambda_c$	The shifting of resonance peak centroid	Monitoring molecular binding events
$\Delta\lambda_s$	The shifting of resonance peak center wavelength	Monitoring molecular binding events
$\Delta FWHM$	The change of resonance peak FWHM	Monitoring molecular binding events
S	The change in the wavelength of the resonance peak for each unit alteration in the sensed Refractive Index	Higher sensitivity means that the sensor could detect smaller changes in the RI
FOM	Figure of merit (based on wavelength)	A higher figure of merit implies that the sensor could detect smaller changes in the refractive index with a narrower spectral peak, indicating better resolution and performance
R	Spectral resolution	The ability of the sensor to distinguish between different wavelengths or colors in the spectrum
LOD	Limit of detection	The smallest concentration or amount of analyte that the sensor could reliably detect and quantify

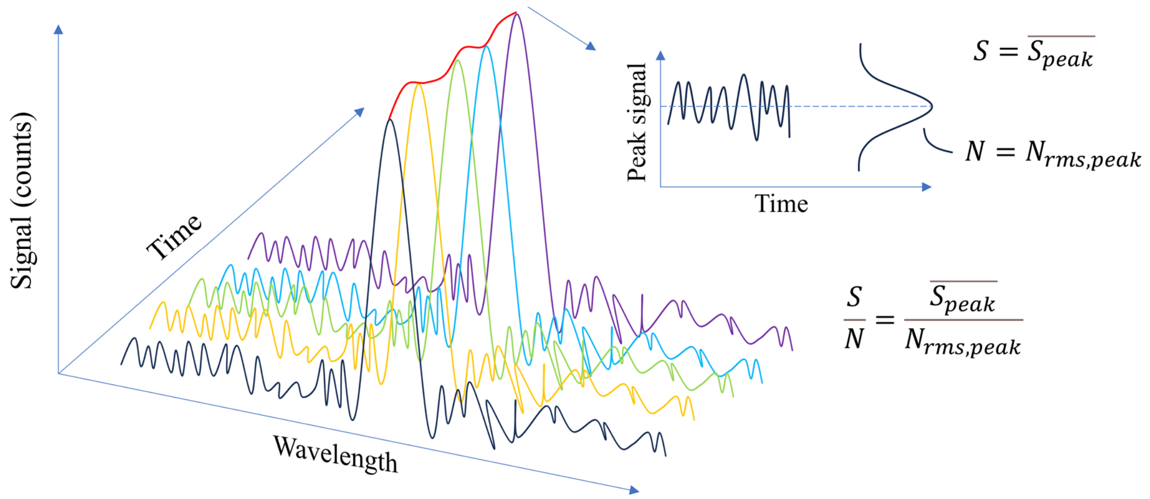

Figure S3. *SNR* calculation. The *SNR* is defined as the average of the peak signal over time divided by the *RMS* noise of the peak signal over the same time.

The *SNR* is defined as the average of the peak signal over time divided by the root mean squared (*RMS*) noise of the peak signal over the same time. In order to get an accurate result for the *SNR* it is generally required to measure over 25 – 50 time samples of the spectrum.

$$\frac{S}{N} = \frac{\overline{S_{peak}}}{N_{rms,peak}} \tag{9}$$

$\overline{S_{peak}}$ means the average of the peak signal over time. $N_{rms,peak}$ means the *RMS* noise of the peak signal over the same time. the *RMS* noise of the peak signal over the same time is calculated as:

$$N_{rms} = \sqrt{\frac{\sum_i^n \left(S_{peak,i} - \overline{S_{peak}}\right)^2}{n}} \tag{10}$$

Where the peak signal over time is measured over n time samples and $\overline{S_{peak}}$ is the average peak intensity, $S_{peak,i}$ is the peak intensity at time i.

In the context of resonance peak wavelength monitoring, the significance of standard deviation is paramount. Standard deviation serves as a statistical measure quantifying the dispersion of data points around the mean within a dataset. In the realm of monitoring resonance peak wavelengths, the magnitude of the standard deviation is indicative of the stability and consistency of the measurement results. To calculate the standard deviation, use the following formula:

$$\sigma = \sqrt{\frac{1}{n}(x_i - \bar{x})^2} \tag{11}$$

Where the one of resonance peak characteristic quantities (e.g. λ_{max}) is measured over n time samples and $\bar{x}$ is the average value of the resonance peak characteristic quantity, x_i is the peak intensity at time i.

To calculate the variance, use the following formula:

$$\sigma^2 = \frac{1}{n}(x_i - \bar{x})^2 \tag{12}$$

Examining the reproducibility of spectral features in replicates or measurements is key to gauging the stability and consistency of signal processing methods. In our assessment of LSPR spectral signal processing robustness, reproducibility emerged as a pivotal metric, revealing high consistency and reliability across multiple experiments. The observed reproducibility underscores the stability of the employed signal processing methods, validating their reliability, and suggesting broad applicability across diverse experimental conditions. This consistency is promising for practical use, indicating that enhancements achieved through signal processing are consistently replicable, enhancing the credibility of our findings and reinforcing the reliability of LSPR spectral signal processing for analytical applications. The reproducibility of a spectrum could usually be expressed by a parameter called the *CV* [9]. In a single sample, with observations x_1, x_2, …, x_n, *CV* is described as:

$$CV(\%) = \left(\frac{s}{\bar{x}}\right) \times 100 \tag{13}$$

Where $\bar{x}$ is the mean value of x_1, x_2, …, x_n.

$$\bar{x} = \frac{1}{n}\sum_{i=1}^{n} x_i \tag{14}$$

$\bar{x} \neq 0$,

and

$$s = \sqrt{\frac{1}{n}\sum_{i=1}^{n}(x_i - \bar{x})^2} \tag{15}$$

are the mean and the standard deviation of the observations, respectively.

S3. LSPR biosensing signal processing strategies integrated on the visual software

The preprocessing method, which includes interpolation and smoothing, enhances the accuracy of LSPR spectra data. Interpolation and smoothing are commonly used techniques in biosensor spectra data processing, and they are of great significance to improve the accuracy and precision of biosensors. Interpolation serves two purposes. (1) Filling in missing data. When the sensor collects data, there may be missing data or sparse data, and interpolation can make the data more complete by estimating the missing data and filling in the gaps. (2) Interpolation can improve spectral resolution. For discrete sensor data, interpolation can be used to generate continuous data, improving spectral resolution, and making the data more granular and closer to reality. Smoothing has three effects. (1) Noise removal. Sensor data is often subject to environmental disturbances and measurement errors. Smoothing can help remove these noises and improve the quality and accuracy of the data. (2) Reducing data fluctuations. Smoothing serves to reduce the pronounced fluctuations in data, enhancing the stability of spectra data and aiding in the accurate capture of trends and patterns in data changes. (3) Improving spectral signal shape. Smoothing can make the shape of the signal smoother and continuous, which helps to accurately identify the feature points and peaks of the spectral signal. Preprocessing methods integrated on visualization software systems are summarized as Table S4.

Table S4. Summary of preprocessing methods integrated in the visual software.

Categories	Method	Advantages	Disadvantages
Interpolation method	Nearest	Simplicity and low computational cost	Lack of smoothness, inaccuracy for gradients, sensitivity to outliers, and limited applicability for continuous data
	Linear interpolation	Simplicity, preservation of linearity applicability to continuous data	Limited accuracy for nonlinear data, inability to capture curvature, lack of smoothness
	Lagrange	Flexibility, Global Interpolation	Runge's phenomenon, numerical instability
	Cubic Hermite	Smoothness, Interpolation with derivative information, preservation of endpoint conditions	Sensitivity to derivative estimates, difficulty in modifying interpolation, not suitable for all functions
	Spline Interpolation	Smoothness, local control, and preservation of endpoint conditions	Limited applicability for noisy data
Smooth method	Moving average	Simple, and low latency	Weak in preserving signal details
	Median filter	Effective at removing isolated noise points, robust against extreme values	Poor performance in smoothing the overall spectrum, not suitable for continuous fluctuations
	Savitzky-Golay filter	Preserving main features, suitable for handling data with significant fluctuations	May introduce excessive smoothing with higher-order polynomial fitting, requires careful parameter selection
	Wavelet transform	Capable of smoothing data at different scales while preserving details	Computational complexity is high, and the choice of wavelet function and scale parameters needs careful consideration

The spectrometer's resolution is intricately tied to both the grating and the slit parameters. A greater grating line count and a narrower slit enhance resolution, though at the cost of diminished signal strength. Conversely, a broader slit yields a stronger signal but with lower resolution. To reconcile this trade-off between high resolution and signal strength, the interpolation method proves valuable. By employing this method, the spectral signal contour is kept relatively smooth, preserving the integrity of subsequent LSPR sensing performance characterization.

The selection of appropriate interpolation algorithms plays a key role in accurately reducing, smoothing, and filling data discontinuities. Different interpolation algorithms show their own unique advantages and application scenarios. There are five types of interpolation methods integrated by the visual software. (A) Nearest. Chooses the y value corresponding to the x value that is nearest to the current x_i value and sets the interpolated value to the nearest data point. (B) Linear interpolation sets the interpolated values to points along the line segments connecting the x and y data points.

$$L_i(x) = y_i + \frac{y_{i+1} - y_i}{x_{i+1} - x_i}(x - x_i) \tag{16}$$

Where $L_i(x)$ is the line segments connecting the x and y data points; i is the index of data points. (C) Lagrange uses the barycentric Lagrange interpolation algorithm. (D) Cubic Hermite. guarantees that the first derivative of the cubic interpolating polynomials is continuous and sets the derivative at the endpoints to certain values to preserve the original shape and monotonicity of the Y data. (E) Spline interpolation calculate the third-order polynomial with two adjacent points. Polynomials meet the following conditions: The first and second derivatives at point x_j are continuous; polynomials satisfy all data points; and the second derivative of the start and end points is 0. $P_i(x)$ is the third-order polynomial between adjacent points (x_i, y_i) and (x_{i+1}, y_{i+1}).

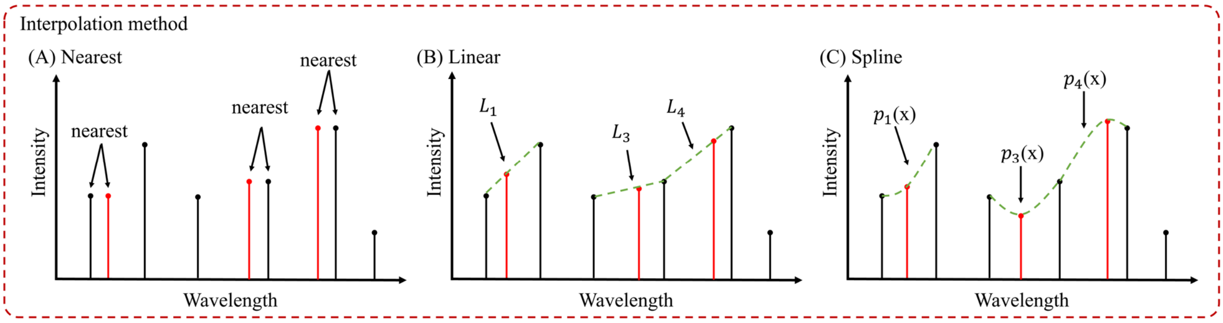

Figure S4. Interpolation method integrated on the integrated visual software. (A) Nearest interpolation. (B) Linear interpolation. (C) Spline interpolation.

When selecting the most suitable interpolation algorithm for LSPR biosensing spectra, spline interpolation is recommended considering that LSPR biosensing spectra are usually continuous and smooth. The reason includes the following four aspects. (1) Spectral continuity requirements. LSPR spectra typically appear as smooth curves, while spline interpolation is known for its smooth nature. Spline interpolation uses low-order polynomial fragments to approximate the curve, which is well adapted to continuity requirements. (2) Accuracy. The spline interpolation method could reproduce the curves in the spectrum more accurately by fitting a lower number of polynomials between adjacent data points. This is critical for the high accuracy required for LSPR spectroscopy. (3) Natural boundary conditions: Spline interpolation allows natural boundary conditions to be set during the interpolation process, ensuring that the interpolation curve behaves well at boundary points, avoiding oscillations or over-approximation that could be introduced by other interpolation algorithms. (4) Smoothness. Spline interpolation produces interpolation curves that not only pass through known data points, but also maintain smooth connections across adjacent fragments, helping to better understand the overall trend of the LSPR spectrum.

The enhancement of signal brightness and the suppression of background noise are the two main factors that determine accuracy and sensitivity. To deal with the noise in LSPR spectral data, the commonly used methods include a variety of spectral smoothing algorithms, which are divided into three categories based on noise frequency, noise statistical characteristics, and waveform patterns. According to different principles, these smoothing algorithms could be divided into three categories [10]: the first is based on noise frequency, such as Fourier transform and wavelet transform [11]; the second is based on noise statistical characteristics, including a variety of filters represented by moving polynomial smoothing filter [12]; and so on; the last category is based on waveform patterns, including mode filtering [13], noise peak discrimination [14], and morphological filtering [15].

Among the many smoothing algorithms, including moving average, Savitzky-Golay smoothing, Loess smoothing, etc., in view of the continuity of the LSPR spectrum and the need to preserve the key features, we choose moving average and Savitzky-Golay smoothing as the preferred algorithm to smooth the LSPR spectral curve more carefully and effectively in subsequent data processing.

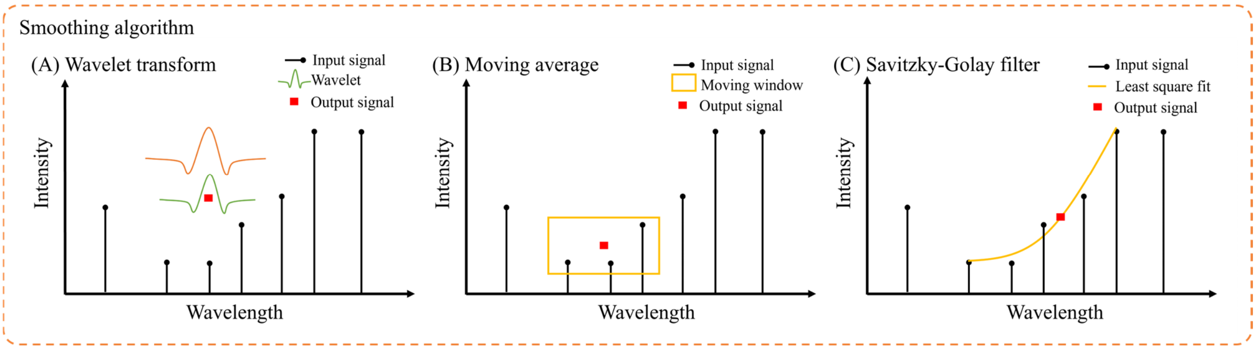

Figure S5. Smoothing algorithms integrated on the visual software. (1) Discrete wavelet transforms. (2) Moving average. (3) Savitzky-Golay filter.

The main reason for using moving averages when collecting spectral data in real time is to instantly reduce transient fluctuations caused by environmental noise or sensor instability, and to improve the stability and readability of real-time data. By calculating the average value within the data window, the moving average helps to smooth out high-frequency noise in the short term, making the spectral data collected in real time more stable. This pre-processing step makes it easier to identify and respond to potential spectral changes during real-time monitoring, increasing confidence in real-time data.

Moving averages are a simple and intuitive smoothing method that smooths the spectrum by calculating the average of data points within a data window. It is suitable for stationary spectral data and has a good removal effect on high frequency noise but may not be able to retain the spike characteristics well. The boxcar width represents the sliding width of the moving polynomial smoothing filter, a moving average filter works by averaging multiple points in the input spectra signal amplitude to generate each point in the output spectra signal amplitude. Repeat this process so that the moving window of M points is used to calculate the average of the dataset.

$$y_i = \begin{cases} \frac{\sum_{j=0}^{M-1} s_{i+j}}{M}, if\ i > 0\ and\ i < N - (M-1) \\ 0, otherwise \end{cases} \quad (17)$$

Where s_{i+j} is the input spectra signal amplitude at wavelength index i, y_i is the output spectra signal amplitude at wavelength index i, M is the average number of spectra data points, and N is the total number of spectra data points.

The reason for the use of Savitzky-Golay smoothing in the spectral post-processing stage is to smooth the entire spectral curve in greater detail, retain the main features in the spectrum and remove high-frequency noise. Savitzky-Golay smoothing is a smoothing method based on polynomial fitting that smooths the spectral curve by performing polynomial fitting locally, helping to remove low-frequency noise over a long period of time and preserve the overall trend of the spectrum. This smoothing method is applied in the post-processing stage to further optimize the overall quality of the spectrum while maintaining important features in the spectrum, improving the interpretability and readability of the data.

Savitzky-Golay filter [16] is one of the most widely used methods. This method is like the moving average method, which is based on window smoothing, but it uses the polynomial least square method to fit the data in the window. The calculation formula is as follows.

$$y(i) = a_0 + a_1 i + a_2 i^2 + \cdots a_k i^k = \sum_{n=0}^{k} a_n i^n \, , |i| \le m \tag{18}$$

Where k represents polynomial order; a_n denotes the coefficients of polynomial; m is the half-width of the smoothing window b and b is equal to $2m+1$. The least-squares criterion is applied to acquire the coefficients a_n as follows:

$$\frac{\partial}{\partial a_n}\left[\sum_{i=-m}^{m} (y(i) - s_i)^2\right] = 0 \tag{19}$$

Where s_i is the input spectra signal amplitude at wavelength index i, result in $k + 1$ simultaneous equation for calculating the unknown coefficients a_n. If we calculate $y(i)$ at $i=0$ and can only acquire a a_0, in the same way, we can get a a_1, a_2 …a_n, Thus, we deduce an expression as follows:

$$y_i = \sum_{j=-m}^{m} a_j^{(0)} s_{i+j} \tag{20}$$

Where y_i is the output of S–G filter at wavelength index i.

The combination of moving average and Savitzky-Golay smoothing could not only stabilize the data to cope with noise in real-time acquisition, but also smooth the spectral curve in a finer way in post-processing, providing a more reliable basis for data analysis. Such a combined processing strategy helps to efficiently process spectral data in a dynamic environment, improving data quality and confidence in analysis.

The method called scans to average is used to average multiple sets of spectral data before and after according to the spectral time series, to eliminate the light intensity fluctuation of the spectrum, and random noise, and help to improve the detection accuracy of λ_{max}. Continuous scanning of the average spectrum also offers the benefits of enhanced signal-to-noise ratio, improved precision, and smoother spectral curves. This approach involves multiple scans that are averaged, resulting in a more reliable measurement with higher quality data. It is particularly

valuable for detecting weak signals, minimizing system drift, and ensuring greater stability in various applications requiring high-quality spectral information.

In addition to various spectral smoothing algorithms that could be used for spectral noise reduction, baseline correction is also one of the commonly used methods. There are three types of commonly used baseline correction methods, one is based on polynomial fitting, which finds the boundary points by calculating the peak range, and then performs background fitting according to these data points, mainly including iterative polynomial [17] piecewise fitting algorithm [18], penalty least squares method [19]. The other type is based on frequency acquisition, which needs to set the filter cut-off frequency, mainly including Fourier transform[20] and wavelet transform [21]: morphological methods [22] could also be used, but because the selection of structural elements is more difficult, the scope of application is smaller than the first two cases.

Zhang et al. [23] proposed a full-spectrum baseline correction method based on the generalized Whittaker smoother, which first used the wavelet transform to obtain the peak range, and then used the smoother to fit, and obtained good results. Based on Zhang's algorithm, a new baseline correction method is proposed by integrating wavelet transform and derivative method. This optimization addresses the overfitting issue present in the original algorithm and improves the accuracy of identifying the characteristic peak region. The algorithm is as follows.

After dividing the spectrum into the characteristic peak region and other regions, different weights are set for the two regions, and the baseline data could be obtained by using the weights for calculation, and the pseudocode is shown in algorithm 1 of Table S5.

Table S5. The workflow of baseline correction.

Algorithm1 Baseline Correction
Input: Spectrum $S = \{(x_1 . y_1), (x_2, y_2), \ldots, (x_n, y_n)\}, scale, wavelet1, wavelet2$
Output: Background result B
$[P, L, R] = Widthchoose(S, scale, wavelet1, wavelet2)$;
Get smooth spectrum S through Savitzky-Golay Filter;
Initialize B;
while exist $(B < S)$
$B(B < S) = S$;
Get baseline B through whittaker smoother;
$S = DivideArea(S, P)$;
for S_i in S:
if S_i in P:
Calculate Overlapping Peaks;
Update S_i;
Get baseline B_i;
Return B:

- **Procedure for the baseline correction**:

1. Calculations were performed using the range of characteristic peak.
2. The data was smoothed and the Savitzky-Golay filter was used to remove the noise.
3. Setting $\lambda = 1$, using the generalized Whittaker smoother for the initial fitting to obtain the background.
4. The part of the background that is smaller than the original spectrum is covered with the original spectrum, and the background is obtained by refitting.
5. For background refinement. The spectrum is divided into multiple regions according to the range of characteristic

peaks, and the calculation is performed for each region: (a) if the current region is a characteristic peak region, according to the range data of the characteristic peaks P, determine whether the current peak P_i is part of the overlapping peak, if so, find all the peaks contained in the overlapping peaks, combine the data, and update the information of the current characteristic peaks P_i and the characteristic peak dataset P; (b) use the generalized Whittaker smoother to calculate the baseline and connect it with the calculated baseline.

Reference

1. Chinowsky, T.M.; Jung, L.S.; Yee, S.S. Optimal Linear Data Analysis for Surface Plasmon Resonance Biosensors. *Sens. Actuators B Chem.* **1999**, *54*, 89–97, doi:10.1016/S0925-4005(98)00316-5.
2. Huang, T.; Wang, J.; Duan, F.; Jiang, J.; Fu, X.; Xu, X. A Simple Algorithm for the Implementation of Second-Order-Polynomial Based Peak-Tracking Methods. *Opt. Fiber Technol.* **2019**, *47*, 192–196, doi:10.1016/j.yofte.2018.10.009.
3. Rodrigues, M.S.; Pereira, R.M.S.; Vasilevskiy, M.I.; Borges, J.; Vaz, F. NANOPTICS: In-Depth Analysis of NANomaterials for OPTICal Localized Surface Plasmon Resonance Sensing. *SoftwareX* **2020**, *12*, 100522, doi:10.1016/j.softx.2020.100522.
4. Thirstrup, C.; Zong, W. Data Analysis for Surface Plasmon Resonance Sensors Using Dynamic Baseline Algorithm. *Sens. Actuators B Chem.* **2005**, *106*, 796–802, doi:10.1016/j.snb.2004.09.032.
5. Jeon, J.; Uthaman, S.; Lee, J.; Hwang, H.; Kim, G.; Yoo, P.J.; Hammock, B.D.; Kim, C.S.; Park, Y.-S.; Park, I.-K. In-Direct Localized Surface Plasmon Resonance (LSPR)-Based Nanosensors for Highly Sensitive and Rapid Detection of Cortisol. *Sens. Actuators B Chem.* **2018**, *266*, 710–716, doi:10.1016/j.snb.2018.03.167.
6. Gul, M.U.; Kadir, K.; Azman, H.K.; Iqbal, S. Detection of R-Peaks Using Single-Scale Wavelet Transform. In Proceedings of the 2019 13th International Conference on Mathematics, Actuarial Science, Computer Science and Statistics (MACS); IEEE: Karachi, Pakistan, December 2019; pp. 1–5.
7. Dos Santos, P.S.S.; Mendes, J.P.; Dias, B.; Pérez-Juste, J.; De Almeida, J.M.M.M.; Pastoriza-Santos, I.; Coelho, L.C.C. Spectral Analysis Methods for Improved Resolution and Sensitivity: Enhancing SPR and LSPR Optical Fiber Sensing. *Sens.* **2023**, *23*, 1666, doi:10.3390/s23031666.
8. Paul, D.; Biswas, R. Highly Sensitive LSPR Based Photonic Crystal Fiber Sensor with Embodiment of Nanospheres in Different Material Domain. *Opt. Laser Technol.* **2018**, *101*, 379–387, doi:10.1016/j.optlastec.2017.11.040.
9. Santos, C.; Dias, C. Note on the Coefficient of Variation Properties. *BRAZ. ELECTRON. J. OF MATH.* **2021**, *2*, 101–111, doi:10.14393/BEJOM-v2-n4-2021-58062.
10. Çataltaş, Ö.; Tutuncu, K. A Review of Data Analysis Techniques Used in Near-Infrared Spectroscopy. *Eur. J. Sci. Technol.* **2021**, doi:10.31590/ejosat.882749.
11. Shao, L.; Lin, X.; Shao, X. A Wavelet Transform and Its Application to Spectroscopic Analysis. *Appl. Spectrosc. Rev.* **2002**, *37*, 429–450, doi:10.1081/ASR-120016391.
12. Savitzky, Abraham.; Golay, M.J.E. Smoothing and Differentiation of Data by Simplified Least Squares Procedures. *Anal. Chem.* **1964**, *36*, 1627–1639, doi:10.1021/ac60214a047.
13. Milojković-Opsenica, D.; Ristivojević, P.; Andrić, F.; Trifković, J. Planar Chromatographic Systems in Pattern Recognition and Fingerprint Analysis. *Chromatographia* **2013**, *76*, 1239–1247, doi:10.1007/s10337-013-2423-9.
14. Leung, A.K.; Chau, F.; Gao, J. A Review on Applications of Wavelet Transform Techniques in Chemical Analysis: 1989–1997. *Chemom. Intell. Lab. Syst.* **1998**, *43*, 165–184, doi:10.1016/S0169-7439(98)00080-X.
15. Zhang, X.; Qi, W.; Cen, Y.; Lin, H.; Wang, N. Denoising Vegetation Spectra by Combining Mathematical-Morphology and Wavelet-Transform-Based Filters. *J. Appl. Remote Sens.* **2019**, *13*, 1, doi:10.1117/1.JRS.13.016503.
16. Zhang, G.; Hao, H.; Wang, Y.; Jiang, Y.; Shi, J.; Yu, J.; Cui, X.; Li, J.; Zhou, S.; Yu, B. Optimized Adaptive Savitzky-Golay Filtering Algorithm Based on Deep Learning Network for Absorption Spectroscopy. *Spectrochim. Acta, Part A* **2021**, *263*, 120187, doi:10.1016/j.saa.2021.120187.
17. Kwiatkowski, A.; Gnyba, M.; Smulko, J.; Wierzba, P. Algorithms of Chemicals Detection Using Raman Spectra. *Metrol. Meas. Syst.* **2010**, *17*, 549–559, doi:10.2478/v10178-010-0045-1.
18. Zhang, F.; Tang, X.; Tong, A.; Wang, B.; Wang, J. An Automatic Baseline Correction Method Based on the Penalized Least Squares Method. *Sens.* **2020**, *20*, 2015, doi:10.3390/s20072015.
19. Ruckstuhl, A.F.; Jacobson, M.P.; Field, R.W.; Dodd, J.A. Baseline Subtraction Using Robust Local Regression Estimation. *J. Quant. Spectrosc. Radiat. Transfer* **2001**, *68*, 179–193, doi:10.1016/S0022-4073(00)00021-2.
20. Jiang, X.Q.; Blunt, L.; Stout, K.J. Development of a Lifting Wavelet Representation for Surface Characterization. *Proc. R. Soc. Lond., A: Math. Phys. Eng. Sci.* **2000**, *456*, 2283–2313, doi:10.1098/rspa.2000.0613.
21. Hoang, V.D. Wavelet-Based Spectral Analysis. *TrAC, Trends Anal. Chem.* **2014**, *62*, 144–153, doi:10.1016/j.trac.2014.07.010.
22. Perez-Pueyo, R.; Soneira, M.J.; Ruiz-Moreno, S. Morphology-Based Automated Baseline Removal for Raman Spectra of Artistic Pigments. *Appl. Spectrosc.* **2010**, *64*, 595–600, doi:10.1366/000370210791414281.
23. Zhang, Z.-M.; Chen, S.; Liang, Y.-Z.; Liu, Z.-X.; Zhang, Q.-M.; Ding, L.-X.; Ye, F.; Zhou, H. An Intelligent Background-Correction Algorithm for Highly Fluorescent Samples in Raman Spectroscopy: Background-Correction Algorithm for Highly Fluorescent Samples in Raman Spectroscopy. *J. Raman Spectrosc.* **2010**, *41*, 659–669, doi:10.1002/jrs.2500.
